# Self-Sustained Regulation or Self-Perpetuating Dysregulation: ROS-dependent HIF-YAP-Notch Signaling as a Double-Edged Sword on Stem Cell Physiology and Tumorigenesis

**DOI:** 10.3389/fcell.2022.862791

**Published:** 2022-06-14

**Authors:** Chin-Lin Guo

**Affiliations:** Institute of Physics, Academia Sinica, Taipei, Taiwan

**Keywords:** ROS, HIF, YAP, notch, tissue mechanics

## Abstract

Organ development, homeostasis, and repair often rely on bidirectional, self-organized cell-niche interactions, through which cells select cell fate, such as stem cell self-renewal and differentiation. The niche contains multiplexed chemical and mechanical factors. How cells interpret niche structural information such as the 3D topology of organs and integrate with multiplexed mechano-chemical signals is an open and active research field. Among all the niche factors, reactive oxygen species (ROS) have recently gained growing interest. Once considered harmful, ROS are now recognized as an important niche factor in the regulation of tissue mechanics and topology through, for example, the HIF-YAP-Notch signaling pathways. These pathways are not only involved in the regulation of stem cell physiology but also associated with inflammation, neurological disorder, aging, tumorigenesis, and the regulation of the immune checkpoint molecule PD-L1. Positive feedback circuits have been identified in the interplay of ROS and HIF-YAP-Notch signaling, leading to the possibility that under aberrant conditions, self-organized, ROS-dependent physiological regulations can be switched to self-perpetuating dysregulation, making ROS a double-edged sword at the interface of stem cell physiology and tumorigenesis. In this review, we discuss the recent findings on how ROS and tissue mechanics affect YAP-HIF-Notch-PD-L1 signaling, hoping that the knowledge can be used to design strategies for stem cell-based and ROS-targeting therapy and tissue engineering.

## Introduction

The ability to self-renew and the potential to differentiate, at least, into one type of mature cell have made stem cells an essential element at various stages of development and a promising tool for regenerative medicine. In general, the selection of stem cell fate depends on the interplay of intracellular signaling and extracellular niche factors. These niche factors can be specified into two groups: chemical molecular factors and physical-mechanical factors. The chemical factors include molecular oxygen (O_2_), reactive oxygen species (ROS), cell metabolites, morphogens, cytokines, growth factors, and extracellular matrix (ECM) molecules. The physical factors contain passive elements (e.g., stiffness, plasticity, viscoelasticity, and 3D topology) and active mechanical forces (created by the cells and the surrounding environment, e.g., compression, stretching, hydrodynamic flow, hydrostatic pressure, and gravity). The responses to these mechano-chemical factors, such as hypoxic responses, cell mechanotransduction, and ROS signaling, have gained growing interest, as accumulating lines of evidence indicated that their interplay is involved in the regulation of stem cell homeostasis and development. Furthermore, the interplay of these responses can lead to tumorigenesis in the presence of genomic instability and aberrant cell signaling. In particular, ROS, the byproduct of energy production that has once been considered harmful due to their ability to damage DNA and proteins, are now recognized as an important signaling factor for the regulation of pathways involved in stem cell physiology and tumor progression.

ROS can spontaneously be created in the natural environment. For living systems, ROS are mainly produced by the mitochondria (Murphy, 2009; Juan et al., 2021) and the membrane-bound NADPH oxidases (NOX) (Bedard and Krause, 2007; Ushio-Fukai, 2009). The production of ROS is primarily controlled by cell metabolism, O_2_, ROS themselves, and several signaling events of niche factors. Examples of these signaling events include the signaling for transforming growth factor-β (TGF-β) (Hiraga et al., 2013; Yan et al., 2014; Watson et al., 2016; Yazaki et al., 2021), epidermal growth factor (EGF) (Azimi et al., 2017; Dustin et al., 2020), insulin (Besse-Patin and Estall, 2014), insulin-like growth factor-1 (IGF-1) (Kang et al., 2016), inflammatory and immune-regulatory cytokines such as angiotensin II and tumor necrosis factor-alpha (TNF-α) (Anilkumar et al., 2008), calcium (Gorlach et al., 2015; Feno et al., 2019), mechanotransduction (Sauer et al., 2008; Brandes et al., 2014a), integrin-ECM interactions (de Rezende et al., 2012; Eble and de Rezende, 2014), and cell–cell adhesions (Lim et al., 2008). Conversely, ROS modulate the activities of several cell fate-decision factors. These factors include the oxygen sensor hypoxia-inducible factor (HIF) (Gerald et al., 2004; Saito et al., 2015; Kobayashi et al., 2021), the mechano-transducer Yes-associated protein (YAP) (Cho et al., 2020), the transducer for the cell differentiation transcription factor Notch, Notch intracellular domain (NICD) (Cai W.-X. et al., 2014; Caliceti et al., 2014; Yan et al., 2014; Sprouse et al., 2019; Yazaki et al., 2021), and the immune suppressor programmed death ligand-1 (PD-L1) (Bailly, 2020). Herein, HIF, YAP, and NICD act as a triad in stem cell physiology and tumorigenesis, as they can physically associate to influence each other (Qiang et al., 2012; Hu et al., 2014; Manderfield et al., 2015; Totaro et al., 2018a; Zhang X. et al., 2018; Engel-Pizcueta and Pujades, 2021). These associations include the coupling between the α subunits of HIF (i.e., HIF-1α/HIF-2α) and YAP (Xiang et al., 2015; Ma et al., 2017; Zhao et al., 2020) and the coupling between YAP and Notch (Totaro et al., 2018a). As for PD-L1, it is the ligand for the immune checkpoint receptors, programmed death-1 (PD-1) (Noman et al., 2014; Janse van Rensburg et al., 2018; Mansour et al., 2020). Recent studies indicate that the expression of PD-L1 is coupled with YAP, Notch, and HIF-1alpha signaling to potentiate the immune suppression and evasion in the progression of tumors (Barsoum et al., 2014; Noman et al., 2014; Lee et al., 2017; Miao et al., 2017; Kim M. H. et al., 2018; Zhou et al., 2019; Wen et al., 2020). Through these couplings, negative and positive feedback regulations can likely be established in the ROS-dependent YAP-HIF-Notch-(PD-L1) signaling axis, leading to a differential or switch-like behavior in the decision of cell fate. Thus, the interplay of hypoxic responses, ROS signaling, and cell mechanotransduction acts as a double-edged sword at the interface of organ development, tissue homeostasis, and cancer progression.

**FIGURE 1:**
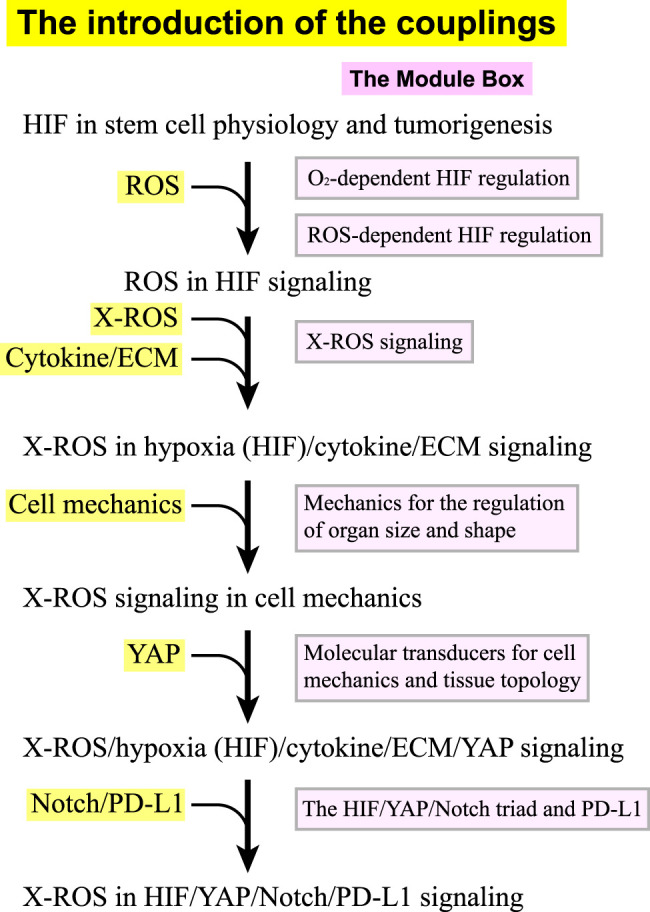
The flow (in black) of introducing the couplings in the X-ROS, HIF, YAP, Notch, and PD-L1 signaling axis. Each coupling is associated with a module box (in pink) in the supporting boxes where detailed information and references can be found.

This review discusses how ROS are involved in the HIF, YAP, and Notch signaling pathways and how their coupling leads to positive or negative feedback for stem cell physiology and tumorigenesis. Given the complexity and the abundant molecular information in the coupling of ROS, HIF, YAP, Notch, PD-L1, and cell–ECM signaling, we organize this review in the following way. We define the signaling in ROS, HIF, YAP, Notch, PD-L1, cell–ECM, and cell mechanics as separated “modules” and introduce/add their coupling one after another. Along with the introduction of the couplings, we provide “module boxes” for each component as the supporting boxes, where detailed molecular–cellular information and references can be found. Figure 1 shows that we start with a brief discussion on HIF signaling in stem cell biology and tumors (Section I), followed by a section on the roles of ROS in HIF signaling (Section II). We then add the coupling of NOX-derived ROS (X-ROS) with the hypoxia (HIF)/cytokine/ECM signaling (Section III), followed by a section on the coupling of X-ROS with cell mechanics (Section IV), where we introduce the functional significance of cell mechanics and mechanotransduction. We then add the coupling of X-ROS/hypoxia (HIF)/cytokine/ECM signaling with YAP signaling (Section V), followed by the final section where we discuss the integrated roles of X-ROS in the HIF/YAP/Notch/PD-L1 signaling (Section VI). In the module boxes, we discuss how molecular oxygen O_2_ regulates HIF stability (Module Box I), how ROS regulate HIF stability (Module Box II), and the X-ROS signaling (Module Box III). A modeling section is provided to discuss the phase diagram of ROS production quantitatively (Math Box I). How cell mechanics regulate organ size and shape (Module Box IV), the molecular transducers for cell mechanics and tissue topology (Module Box V), and the coupling of HIF/YAP/Notch triad with PD-L1 (Module Box VI) are also addressed.

## Main Text

### The Roles of HIF in Stem Cell Physiology and Tumorigenesis

**FIGURE 2:**
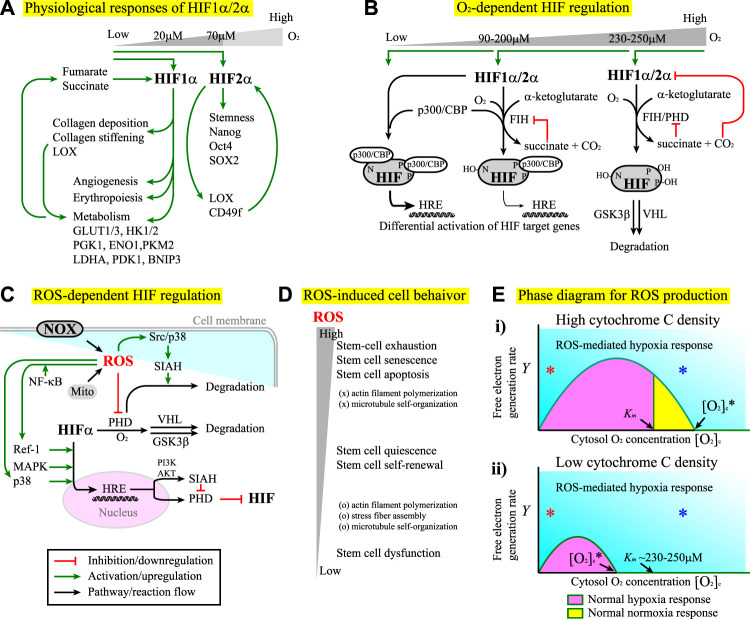
**(A)** The differential responses of HIF-1α and HIF-2α to hypoxia conditions. See the main text for details. For all the figures hereafter, red lines indicate inhibition or downregulation, green lines indicate activation or upregulation, blue lines indicate physical association or recruitment, and black lines indicate the flow of the pathways, cascades, or reaction. **(B)** The factor inhibiting HIF (FIH) and prolyl hydroxylase domain-containing proteins (PHD) regulate HIF-1α and HIF-2α stability and transcriptional activity in an O_2_ concentration-dependent manner. See Module Box II for details. **(C)** ROS produced by the NOX and/or the mitochondria (Mito) exhibit both positive and negative effects on the regulation of HIF-α subunits. See Module Box III for details. **(D)** Stem cells exhibit differential phenotypical behavior and cytoskeletal dynamics in response to the changes in ROS concentrations. **(E)** The phase diagrams for the separation of ROS-mediated hypoxia responses (cyan) from normal hypoxia (pink) and/or normoxia (yellow) responses at **(i)** high and **(ii)** low cytochrome C densities corresponding to the high and low critical oxygen concentrations ([O_2_]_c_*) for the onset of ROS-mediated hypoxia responses, respectively. See Math Box I for details. *K*_m_ is the *K*_m_ value of PHD for [O_2_] association (∼230–250 μM (Fong and Takeda, 2008)). The *x*-axis indicates the cytoplasmic oxygen concentrations (in arbitrary units). The *y*-axis indicates the free-electron generation rate in the electron transfer chain (in arbitrary units). Red * and blue * indicate that ROS-mediated hypoxia responses can occur at low and high (even above *K*_m_) oxygen concentrations, respectively, as long as the free electron generation rate *y* is sufficiently high.

For stem cell applications, one important issue is to maintain the full pluripotency of stem cells, which often requires hypoxia conditions. The major cellular responses to hypoxia are primarily mediated by hypoxia-inducible factors (HIFs) which act as transcription factors (Ezashi et al., 2005). HIFs consist of one α subunit and one β subunit. While the β subunit is expressed constitutively, the α subunit is regulated in an O_2_- and ROS-dependent manner (Module Boxes I and II). Under normoxia, the α subunits are continuously ubiquitinated and targeted to degradation. Under hypoxia, the α subunit is stabilized to form a dimer with the β subunit. By translocating to the nucleus, the dimer regulates downstream gene expression through binding to the hypoxia-responsive element (HRE) (Harris, 2002). Three forms of α subunits, HIF-1α, HIF-2α, and HIF-3α, have been identified (Wang et al., 1995; Tian et al., 1997; Xu and Li, 2021). While HIF-1α and HIF-2α are structurally similar and share functions to a certain extent, HIF-3α contains several splice variants, some of which act as dominant-negative inhibitors of HIF-1α or HIF-2α (Majmundar et al., 2010; Xu and Li, 2021). Under hypoxia, HIF-1α induces transcription of more than 60 genes to regulate responses such as erythropoiesis, angiogenesis, cell proliferation, cell survival, and glucose and iron metabolism. By doing so, HIF-1α promotes oxygen delivery to the hypoxic region (Semenza, 2003) and switches cells to glycolytic metabolism in response to hypoxia (Lee J.-W. et al., 2004). HIF-1α also induces the expression of genes responsible for collagen deposition and stiffening (Gilkes et al., 2013), one of which is the gene for lysyl oxidase (LOX), the enzyme crosslinking ECM (Ji et al., 2013). In addition, through the altered metabolic flux that promotes the hydroxylation of collagen, HIF-1α renders the collagen matrix more resistant to degradation (Stegen et al., 2019). ECM stiffening, in turn, further promotes metabolic reprogramming (Ge et al., 2021). It has been shown that the altered cell metabolism can potentially activate HIF-1 (Halligan et al., 2016), leading to a positive feedback cycle. Consequently, niche stiffening and niche hypoxia can act synergistically through HIF-alpha to promote a bifurcated selection of cancer cell fate between the apoptotic and the aggressive phenotypes (Lv et al., 2017). In comparison, HIF-2α primarily regulates the expression of a panel of embryonic transcription factors and stemness-related genes such as OCT4, NANOG, and SOX2 (Covello et al., 2006; Gordan et al., 2007; Hu et al., 2014; Petruzzelli et al., 2014). Nevertheless, there are lines of evidence showing that HIF-2α also participates in ECM remodeling. For example, HIF-2α induces the expression of LOX to accelerate ECM deposition and crosslinking in thyroid-associated orbitopathy (Hikage et al., 2019) and the expression of laminin receptor CD49f to facilitate stem cell development in germline stem cells (GSCs), where the expression of CD49f further enhances the expression of HIF-2α, thereby forming a positive feedback loop (Au et al., 2021) (Figure 2A).

The segregation of biological functions in HIF-1α and HIF-2α signaling makes it plausible that these two factors are stabilized under different hypoxia conditions (Hu et al., 2014). HIF-1α is stabilized under severe hypoxia (niche oxygen concentrations <2%, i.e., [O_2_] < 20 μM) (Hu et al., 2014). In comparison, HIF-2α is stabilized in a wider range of oxygen concentrations: from physiological oxygen concentrations (∼7%, i.e., [O_2_] ∼70 μM) to severely low oxygen concentrations (<2%) (Hu et al., 2014). The restricted requirement for HIF-1α stabilization indicates that the upregulation of glycolysis only occurs if the niche oxygen concentration is extremely low. As a result, the cells primarily use oxidative phosphorylation as the major energy production process. In contrast, the fact that HIF-2α is stabilized in a wide range of oxygen concentrations indicates that the cells can robustly maintain certain behavior such as stemness over the fluctuation of niche oxygen, a requisite to sustain cell fate in a fluctuating microenvironment. Note that the restricted conditions for the stabilization of HIF-1α might no longer exist in tumors, allowing tumor cells to use anaerobic metabolism and elicit angiogenesis even with abundant O_2_ in the niche (Semenza, 2003; Masoud and Li, 2015). In fact, both HIF-1α and HIF-2α play important roles in tumor angiogenesis (Krock et al., 2011), survival (Chen and Sang, 2016), proliferation (Hubbi and Semenza, 2015), immune evasion (Barsoum et al., 2014), plasticity (Terry et al., 2017), invasion and metastasis (Zhong et al., 1999), chemo- and radio-therapy resistance (Moeller et al., 2004; Rohwer and Cramer, 2011), pH regulation, and metabolism (Hulikova et al., 2013). These two factors also help the emergence and the maintenance of cancer stem cells (CSCs). The detailed review can be found elsewhere (Heddleston et al., 2010; Schoning et al., 2017; Tong et al., 2018).

### The Roles of ROS in HIF Signaling

*In vivo*, the stability of HIF-α subunits is primarily regulated by molecular oxygen (Module Box I and Figure 2B) and ROS (Module Box II and Figure 2C). Once stabilized, HIF-1α induces the transcription of multiple genes to boost glucose and energy metabolism (Figure 2A). Examples include genes for glucose transporters (e.g., GLUT1 and GLUT3), hexokinase (e.g., HK1 and HK2), pyruvate conversion [e.g., lactate dehydrogenase A (LDHA), pyruvate dehydrogenase kinase (PDK), pyruvate kinase M2 (PKM2), enolase 1 (ENO1)], and mitochondrial autophagy [e.g., BCL2/adenovirus E1B 19 kDa protein-interacting protein 3 (BNIP3)], the detailed review of which can be found elsewhere (Semenza, 2010). The boost of glucose metabolism leads to the accumulation of intermediate-state metabolites, among which α-ketoglutarate (Duran et al., 2013), fumarate (Yang et al., 2012), and succinate (Tannahill et al., 2013), the by-products in the Krebs cycle, can regulate the stability of HIF through the positive or negative modulation on the activity of prolyl hydroxylase domain-containing proteins (PHD), the primary enzyme to destabilize HIF-α subunits (Module Box I and Figure 2B). Consequently, positive and/or negative feedback might exist in the interdependent regulation of HIF-1 activity and metabolic reprogramming. Metabolic reprogramming also occurs in response to ECM stiffening (Ge et al., 2021) through a YAP/TAZ-mediate upregulation of GLUT1/GLUT3 (Cosset et al., 2017; Liu et al., 2020b). The resultant stabilization of HIF-1α can further stiffen ECM (Gilkes et al., 2013), leading to positive feedback in the coupling of hypoxia responses and ECM remodeling (Figure 2A). Moreover, the activity of HIF-1α is sensitive to stressful conditions such as hypercapnia (Selfridge et al., 2016), in which the HIF-1α activity is suppressed, and the host is at the risk of opportunistic infections (Cummins et al., 2014). In fact, tissue hypoxia has a significant impact on inflammatory signaling pathways (Cummins et al., 2016), a part of which depends on ROS (Kohchi et al., 2009; Chen et al., 2016). The term “immunometabolism” for the interdependence of HIF activity and immunity has thus been proposed (Halligan et al., 2016). Besides, ROS is an essential factor for cell functioning and a deleterious factor for mutations, tumorigenesis, and cell apoptosis (Skonieczna et al., 2017). Such a dual role of ROS has been found in the selection of stem cell fate. For example, while ROS at moderately low levels are required to maintain stem cell quiescence and self-renewal, ROS at moderately high levels lead to stem cell proliferation and differentiation (Valle-Prieto and Conget, 2010; Burtenshaw et al., 2017). Consequently, over-suppressing ROS levels impairs stem-cell functioning, and over-elevating ROS levels leads to stem-cell exhaustion, premature aging (senescence), and apoptosis (Schieber and Chandel, 2014) (Figure 2D).

ROS are primarily produced in mitochondria (Murphy, 2009; Juan et al., 2021), where free electrons in the electron transport chain (ETC) are leaked to bind to O_2_ and form superoxide O_2_• (or O_2_^−^) instead of the water molecule. In general, the yield of ROS depends on the generation rate of free electrons (set as *Y*) and the intracellular oxygen concentration (set as [O_2_]_*c*_). To see how a free electron selects to become O_2_^−^ rather than a water molecule, we set up a simple mathematical analysis (Math Box I) and obtained a critical cytoplasmic oxygen concentration [O_2_]_c_*. For [O_2_]_*c*_ above [O_2_]_c_*, the free electrons predominantly select to become ROS. We also obtained the critical electron generation rate ***Y*** *. For ***Y*** above ***Y*** *, over 50% of the free electrons select to become ROS (Figure 2E). In the absence of any feedback or transcriptional regulation, the phase diagram in Figure 2C suggests three scenarios. The first occurs when the critical oxygen concentration [O_2_]_c_* (depends on the density of cytochrome c) is above the *K*_*m*_ of value of PHD for [O_2_]c association (Figure 2E(i)), where PHD is the primary enzyme to destabilize HIF alpha subunits (Module Box I and Figure 2B). For this case, there is a region, *K*_*m*_ ≤ [O_2_]_*c*_ ≤ [O_2_]_c_*, in which PHD promotes the degradation of HIF-α subunits through the association with O_2_ and below which (i.e., [O_2_]_*c*_ < *K*_*m*_) HIF-α subunits are stabilized. When [O_2_]_*c*_ > [O_2_]_c_*, PHD is deactivated by ROS through, for example, cysteine oxidation (Module Box II and Figure 2C), and hence, HIF-α subunits are stabilized. Such a scenario leads to a “pathological” hypoxia response under hyperoxia conditions; in other words, the oxygen concentration is above normoxia, but HIF signaling is activated. The second scenario occurs when [O_2_]_c_* is less than *K*_*m*_ (Figure 2E(ii)). In this case, PHD is always deactivated by ROS even for [O_2_]_*c*_ > *K*_*m*_, the region where PHD is supposed to promote the degradation of HIF-α subunits. This scenario allows cells to maintain HIF signaling over a wide range of niche oxygen concentrations, which might be used for robust control of stem cell fate or for aberrant cellular behavior (such as tumorigenesis and cancer stemness). The third is that ROS-mediated hypoxia response can always occur at extremely low and high oxygen concentrations (Figure 2E, red * and blue *, respectively), as long as the yield of free electrons by cell metabolism is sufficiently high (as in the case of tumor or inflammation). This scenario might contribute to the pathological hypoxia responses under normoxia or hyperoxia conditions.

The fact that not only O_2_, but also ROS serve as a HIF regulator might be rationalized by the observation that hypoxia responses, such as those mediated by HIF-2α, are often required for the maintenance of stemness in stem cells (Ezashi et al., 2005; Covello et al., 2006; Keith and Simon, 2007; Mazumdar et al., 2009; Pervaiz et al., 2009; Heddleston et al., 2010; Abdollahi et al., 2011; Hu et al., 2014; Petruzzelli et al., 2014). Having ROS as an additional regulator might help cells to maintain a robust control on stemness against the niche oxygen fluctuation. The ability to use ROS as an additional regulator allows cells to maintain a robust control on stemness against the niche oxygen fluctuation. Regarding the interplay of ROS and hypoxia responses, we should point out that there are both positive and negative feedback regulations. To maximize the usage of O_2_ as the major energy resources, the yield of free electrons from cell metabolism must fit the availability of O_2_ in the niche. A high yield of free electrons demands more O_2_ from the niche. Using the leakage of electrons into ROS as a signal, this demand evokes hypoxia responses, as one consequence of HIF-1α signaling is to induce angiogenesis (Krock et al., 2011) which can enhance O_2_ delivery to the niche. Enhanced delivery of O_2_, however, might not cope with the demand of removing abundant free electrons but instead produce more ROS. In addition, hypoxia responses include upregulating the expression of oxygen carriers and glycolytic enzymes (Hu et al., 2014). Such effects lead to higher intracellular oxygen concentrations (by the abundance of carriers) and larger free-electron generation rates (by excessive glycolysis), hence creating a positive feedback loop to couple sustained ROS production and hypoxia responses into a vicious cycle. Fortunately, prolonged HIF-1α signaling increases the expression of ROS scavengers, prolyl hydroxylase domain-containing protein 2 (PHD2), and the factor inhibiting HIF 1 (FIH-1), which promote HIF-1α degradation (Kobayashi et al., 2021) and cease HIF signaling as negative feedback control.

The existence of positive and negative feedback provides a possibility of bifurcation and suggests that ROS signaling can be a double-edged sword (Saito et al., 2015; Di Meo et al., 2016). Under normal conditions, cells use the multiplex versatility of ROS-mediated hypoxia responses to adapt to or cope with niche fluctuations (Pervaiz et al., 2009; Valle-Prieto and Conget, 2010), thereby maintaining stem cell physiology and cell fate in a robust manner. In the abnormal situations such as tumorigenesis, tumor cells take advantage of ROS-mediated hypoxia responses to promote cancer stemness, invasiveness, drug resistance, and immune evasion (Keith and Simon, 2007; Mazumdar et al., 2009; Heddleston et al., 2010; Barsoum et al., 2014; Peng and Liu, 2015; Aponte and Caicedo, 2017; Schoning et al., 2017; Yeo et al., 2017; Tong et al., 2018). ROS can also cause stem cell exhaustion and premature aging (Turrens, 2003; Schieber and Chandel, 2014; De Gaetano et al., 2021) (Figure 2D). The onset of these physiological and pathological processes is certainly cell- and tissue-context dependent and could be differential or switch-like. In fact, switch-like behavior with a multi-stability has been reported in the ROS regulation of human cells (Huang JH. et al., 2021). Elucidating how these processes occur requires a molecular–cellular understanding of the interplay between ROS and other signaling pathways.

### The Coupling of NOX-Derived ROS (X-ROS) With Hypoxia/Cytokine/ECM Signaling

Apparently, molecular oxygen is not the only niche factor regulating stem cell physiology. Other factors include ECM molecules and cytokines such as TGFβ1, bone morphogenic protein (BMP), angiotensin II (Ang II), platelet-derived growth factor (PDGF), EGF, and IGF-1. Similarly, mitochondria are the only source of producing ROS. Other sources include membrane-bound NADPH oxidases (NOX) (Bedard and Krause, 2007), cytochrome p450 (Veith and Moorthy, 2018), xanthine oxidase (XO) (Battelli et al., 2016b; a), and nitric oxide synthase (NOS) (Wilkinson-Berka et al., 2013; Di Meo et al., 2016). Among them, NOX are known to regulate the differentiation and self-renewal of stem cells and potentiate the self-renewal, metastasis, and drug resistance of CSCs through, for example, Notch, mitogen-activated protein kinases (MAPKs, including Erk1/2, Jun N-terminal kinase (JNK), and p38 kinase), and phosphatidyl-inositol-3-kinase- (PI3K-) AKT signaling (Skonieczna et al., 2017). Crosstalk between NOX and the signaling of TGFβ1 (Ning et al., 2002), BMP (Sanchez-De-Diego et al., 2019), Ang II (Nguyen Dinh Cat et al., 2013), PDGF (Al-Eisa and Dhaunsi, 2017), EGF (Weng et al., 2018), and IGF-1 (Xi et al., 2013; Kang et al., 2016) has also been reported and/or reviewed. Moreover, NOX potentiate the interaction between ECM and cell surface receptors such as integrin β1 (Heo and Lee, 2011), thereby facilitating cell adhesion and migration, particularly in the presence of niche cytokine factors such as IGF-1 (Chiarugi et al., 2003; Meng et al., 2008; Heo and Lee, 2011; Xi et al., 2013).

**FIGURE 3:**
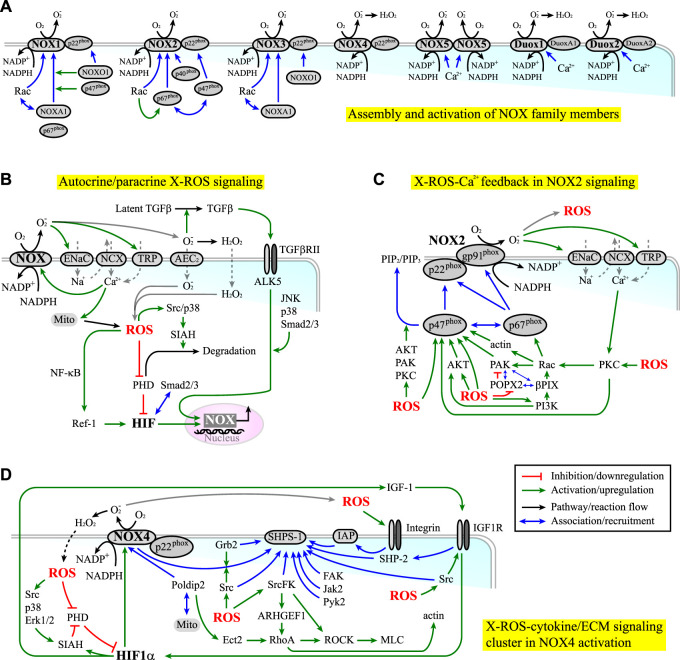
**(A)** The assembly (blue lines) and activation (green lines) of NOX family members. **(B)** NOX-derived ROS (X-ROS) signaling can elicit both autocrine and paracrine effects. Red lines indicate inhibition or downregulation. Green lines indicate activation or upregulation. Blue lines indicate physical association or recruitment. Black lines indicate the flow of the pathways or a reaction. **(C)** NOX2 signaling is coupled with cytoskeleton dynamics and kinase/phosphatase activities through an X-ROS-Ca^2+^ feedback loop. **(D)** NOX4 signaling is associated with the clustering of signaling molecules involved in the cytokine/integrin-ECM signaling.

NOX can produce ROS in the extracellular space (e.g., the niche) and inside the cells. Seven members of NOX have been identified, including NOX1-5 and dual oxidase 1-2 (Duox1-2), each of which has its own NOX gp91^phox^ homolog (Brown and Griendling, 2009; Brandes et al., 2014a; b; Fukai and Ushio-Fukai, 2020) (Module Box III and Figure 3A). The ROS produced by NOX in the extracellular space can enter or regulate nearby cells as a paracrine signal through ion exchangers and ion channels, such as anion exchange protein 2 (AEC2) (Ghio et al., 2003) and epithelial sodium channels (ENaC) (Helms et al., 2008; Liu et al., 2016b) (Figures 3B,C). Alternatively, they can liberate latent cytokines stored in the ECM reservoirs, such as TGF-β1 (Watson et al., 2016), leading to a systemic niche remodeling. On the contrary, the ROS produced in the cell can serve as an autonomous signal to induce oxidative stress (Schieber and Chandel, 2014) or hypoxia responses (e.g., through inactivating PHD to elicit HIF-1α/HIF-2α signaling (Figure 2C)). In turn, hypoxia responses mediated by HIF-1α signaling can upregulate the expression of NOX (e.g., NOX4 (Diebold et al., 2010)) and PHD (Kobayashi et al., 2021) (Figure 3B). These lines of evidence suggest that both positive and negative feedback regulations exist in the axis of NOX-ROS-HIF signaling and that NOX act both upstream and downstream of the HIF signaling.

Beyond the HIF-mediated regulation on the expression level, the activities of NOX are modulated by the assembly and the post-translational modifications (PTMs) of their cytoplasmic and membrane subunits. In fact, almost all NOX subunits are subject to functional-relevant PTMs. Such regulation is under the crosstalk with cytokine signaling (e.g., IGF-1, TGF-β1, EGF, and PDGF signaling) and integrin/ECM signaling (including those involved in cell mechanotransduction (Brandes et al., 2014a), cell adhesion (Schroder, 2014), and cell migration (Brown and Griendling, 2009)). Further, these signaling activities are reciprocally modulated by ROS (Ning et al., 2002; Chiarugi et al., 2003; Ali et al., 2006; Block et al., 2008; Meng et al., 2008; Heo and Lee, 2011; Touyz and Briones, 2011; Xi et al., 2013; Brandes et al., 2014a; Jiang et al., 2014; Yan et al., 2014; Gau and Roy, 2018; Pietruczuk et al., 2019; Fukai and Ushio-Fukai, 2020). In the presence of cooperative or synergistic coupling in the NOX/cytokine/ECM signaling, a bistable or multi-stable switch might be established to potentiate the selection of cell fate, as observed in the regulation of ROS (Huang JH. et al., 2021). This scenario is likely to occur in the processes involving NOX1-2 and NOX4, in that NOX2 and NOX4 are reported to involve in stem cell differentiation and self-renewal, and NOX1-2 and NOX4 are found to potentiate CSC growth, survival, and drug resistance (Brandes et al., 2014b; Skonieczna et al., 2017). The regulations of these NOX mainly occur through serine/threonine phosphorylation (NOX1-3) and tyrosine phosphorylation (NOX4) (Rastogi et al., 2016; Skonieczna et al., 2017). In comparison, the regulation of NOX5 and Duox1-2 primarily depends on calcium. This difference is correlated with the fact that most cancers have dysregulated kinase/phosphatase activities. Other than cancers, NOX-derived ROS correlate with diseases such as cardiomyopathies (Prosser et al., 2011; Kerr et al., 2015). The term “X-ROS” has thus been invented to describe the rapid and localized mechano-chemical signaling elicited by “NOX-derived ROS” (Prosser et al., 2011). A brief review of the regulation of NOX 1-4 and a short discussion on how they are coupled with cytokine and ECM signaling can be found in Module Box III.

One example of positive coupling in X-ROS/cytokine/ECM signaling is the potential feedback amplification along the NOX2-ROS-Ca^2+^-protein kinase C (PKC) signaling axis (Module Box III and Figure 3C). Another is the NOX4-ROS-HIF-IGF-1 signaling, which occurs through the clustering of NOX4, steroid receptor coactivator (Src) kinase, Src homology 2- (SH2-) domain-containing protein tyrosine phosphatase (SHP) substrate-1 (SHPS-1), growth factor receptor-bound protein 2 (Grb2), integrin-associated protein (IAP), and growth factor receptors such as IGF-1 receptor (IGF-1R) (Module Box III and Figure 3D). Among them, IAP is a transmembrane protein associated with several integrins. The association of IGF-1R with IAP thus enables the crosstalk between IGF-1R and ECM/integrin signaling, thereby coupling ROS signaling and growth factor stimulation with cell–ECM adhesion and cell migration (Maile et al., 2003). To add more systemic niche effects, NOX4-derived ROS can induce HIF-1α signaling (e.g., through downregulating the PHD activity (Xu and Li, 2021), which in turn upregulates the expressions of NOX4 (Diebold et al., 2010) and IGF-1 (Poon et al., 2009; Prabhakar and Semenza, 2012; Huang et al., 2017), leading to autonomous (through NOX and IGF-1) and non-autonomous effects (through IGF-1 acting on neighboring cells) in X-ROS-HIF-IGF-1 signaling (Figure 3D).

The association of NOX4 and Src kinase within the SHPS-1 scaffold allows Src kinase to phosphorylate NOX4 and enhance ROS production (Xi et al., 2013). Reciprocally, ROS target and oxidize the cysteine residues at the catalytic domain of Src kinase, thereby activating the kinase (Giannoni et al., 2010; Ray et al., 2012; Xu et al., 2017; Heppner et al., 2018; Dustin et al., 2020; Heppner, 2021). Such mutual interplay leads to localized feedback amplification in IGF-1 and integrin/ECM signaling. A detailed review of redox regulation on, for example, IGF-1 signaling can be found elsewhere (Lennicke and Cocheme, 2021a; Lennicke and Cocheme, 2021b). Clearly, with the abundance of cysteine residues in most signaling molecules, Src kinase is not the only substrate sensitive to ROS. The MAPKs (including JNK (Santabarbara-Ruiz et al., 2015), Erk (Aikawa et al., 1997), p38 kinase (Kulisz et al., 2002; Emerling et al., 2005; Lee et al., 2012; Santabarbara-Ruiz et al., 2015), and big MAP kinase (BMK1/Erk5) (Abe et al., 1996), the Ca^2+^/calmodulin-dependent kinase 2 (CaMK2) (Basu et al., 2019), the cGMP-dependent protein kinase or protein kinase G (PKG), the PI3K/AKT (Ray et al., 2012; Koundouros and Poulogiannis, 2018), the PKC (Gong et al., 2015; Steinberg, 2015), the cAMP-dependent protein kinase (PKA (Andre et al., 2013)), and the focal adhesion kinase (FAK) (Ben Mahdi et al., 2000) are redox sensitive and subject to activation by ROS. In parallel, protein serine/threonine phosphatases (PPP, including PP1 (Kim et al., 2015), PP2A (Low et al., 2014; Raman and Pervaiz, 2019), and PP2C-like partner of PIX 2 (POPX2 (Kim P. R. et al., 2020))), and protein tyrosine phosphatases (PTP), including PTP1B, the low molecular weight PTP (LMW-PTP, the major PTP for FAK) (Chiarugi et al., 2003), PTEN (Ray et al., 2012), SHP-2 (Chattopadhyay et al., 2017), and cdc25C (Rudolph, 2005; Seth and Rudolph, 2006; Han et al., 2018)) are also redox-sensitive and can be inhibited by oxidation. Through the ROS-mediated modulation of the kinase and phosphatase activity and the reciprocal phosphorylation-dependent ROS production, it is possible to have positive or negative feedback loops in the ROS-dependent cytokine/ECM signaling. Moreover, the feedback regulation on the expression levels has been reported. For example, ROS-activated p38 kinase and Erk1/2, two key kinases involved in cytokine signaling, can enhance the expressions of NOX (e.g., NOX4 (Diebold et al., 2010)) and the nuclear translocation of HIF-1α (Richard et al., 1999; Sodhi et al., 2000; Flugel et al., 2007). Nuclear HIF-1α, in turn, promotes the expression of seven in absentia homolog 2 (SIAH2), one of the enzymes targeting PHDs for ubiquitin-mediated proteasome degradation (Xu and Li, 2021), in a PI3K/AKT-dependent manner (Koundouros and Poulogiannis, 2018; Perillo et al., 2020). Src and p38 kinases can further phosphorylate and activate SIAH2 (Khurana et al., 2006; Sarkar et al., 2012), thereby forming the positive feedback amplification in HIF-1 signaling (Figures 2B, 3C). In addition, HIF-1α can induce the deposition and stiffening of collagen (Gilkes et al., 2013), and ROS can upregulate the expression of integrins and ECM molecules such as laminin (Liu J. et al., 2016) and fibronectin (Lee H. B. et al., 2004). These effects reinforce the ligand-receptor interactions in the ROS-modulated cytokine and ECM signaling (Lamari et al., 2007; Liu J. et al., 2016).

The preferential coupling of NOX4 with protein tyrosine kinases (PTKs) raises an important issue in stem cell biology. From an evolutionary point of view, PTKs have a specific relation with ROS. PTKS were primarily present in multicellular organisms during the episodes of increasing atmospheric O_2_ concentrations, which drove the use of O_2_ as the major energy resource in multicellular organisms (Dustin et al., 2020). The emergence of NOX in multicellular organisms had evolved at the same time (Kawahara et al., 2007; Holmstrom and Finkel, 2014). Thus, it is reasonable that PTKs are related to cell differentiation and functionalization in multicellular organisms (thus linked to stem cell homeostasis) (Dustin et al., 2020) and that NOX are coupled with RTKs in oxidative phosphorylation, metabolism, and tissue remodeling, as in the case of NOX4 (e.g., through Poldip2, TGFβ, and IGF-1/insulin signaling). In fact, PTKs have been recognized as a major target for clinical treatments (i.e., through tyrosine kinase inhibitors (TKI)) of cancers (Zhang et al., 2009; Dustin et al., 2020). Likewise, NOX have been used as a target for the treatments of, for example, thrombosis, osteoarthritis, diabetes-related complications, stroke, cancers, and pulmonary fibrosis (Bonner and Arbiser, 2012; Hecker et al., 2012; Violi and Pignatelli, 2014; Morel et al., 2015; Zhang et al., 2016; Peng et al., 2019). A HIF-1α/NOX4 signal pathway has been identified to induce drug and radiation resistance in ovarian cancer (Liu W. J. et al., 2021). It would be interesting to investigate whether a combinatory target therapy on NOX and PTKs provides additive or synergistic benefits on diseases such as cancer and systemic diseases.

### The Coupling of X-ROS Signaling With Cell Mechanics

Besides cytokine/ECM signaling, other feedback amplifications in ROS responses include the mitochondria-dependent, ROS-induced ROS release, and the mitochondria-mediated crosstalk between ROS and the calcium flux, a detailed review of which can be found elsewhere (Zorov et al., 2014; Gorlach et al., 2015; Javadov, 2015; Feno et al., 2019). Herein, we focus on the coupling of NOX with cell mechanics and mechanotransduction, an emerging issue in the fields of stem cell research, cell therapy, wound healing, and cancer (Paszek et al., 2005; Kono et al., 2012; Liu et al., 2020a; Wilkinson and Hardman, 2020; Bergert et al., 2021; Hayward et al., 2021). In fact, a great deal of interest has recently been raised in the roles of cell mechanics in the key cellular processes, such as proliferation, cell death, cell differentiation, and cell migration (Chen et al., 1997; Horowitz et al., 1999; Lecuit and Lenne, 2007; Settleman and Baum, 2008; Grosberg et al., 2011), and the maintenance of stem cell pluripotency (Discher et al., 2009; Jaalouk and Lammerding, 2009; Mammoto and Ingber, 2009; Wozniak and Chen, 2009). These key processes often involve molecular–cellular interactions at the boundaries, ranging from the membrane of a single cell to the interfaces between cells and between cells and ECM. Examples include epithelial–mesenchymal interaction (EMI) in the hair follicle (Sick et al., 2006) and tooth (Mammoto et al., 2011) formation, EMI in wound healing (Chong et al., 2009; Seltana et al., 2010), endothelial cell–pericyte interaction in angiogenesis (Gerhardt and Betsholtz, 2003), and endothelial cell–hepatocyte interaction in liver development and regeneration (Inamori et al., 2009). In these examples, the importance of cell mechanics is manifested in the ability of cells to control their size and shape (i.e., 3D topology and geometry) at the interacting boundaries, which in turn profoundly influence the binary decision of cells, for example, to proliferate or differentiate (Folkman and Moscona, 1978; Spiegelman and Ginty, 1983; Piccolo et al., 2014). In line, recent experiments have shown that the fate of stem cells (e.g., self-renewal and differentiation) and the development of organs (such as branching morphogenesis in tubular organs) can be controlled by engineered geometries on the cell–cell and cell–ECM interacting boundaries (Chen et al., 1997; Nelson et al., 2006; Gomez et al., 2010; Silver et al., 2020). Conversely, abnormality or failure in the control of cell size and shape at the interacting boundaries is often found in diseases such as organ malformation, atherosclerosis, cancer, and tumor invasion (Chen et al., 1997; Paszek et al., 2005; Nelson et al., 2006), and cancer-associated fibroblast- (CAF-) aided initiation and maintenance of cancer stemness (Chen et al., 2014). A conceptual discussion on how mechanics contribute to the regulation of cell/organ size and shape can be found in Module Box IV. Reviews on the details of mechano-sensing can also be found elsewhere (Cai et al., 2021).

**FIGURE 4:**
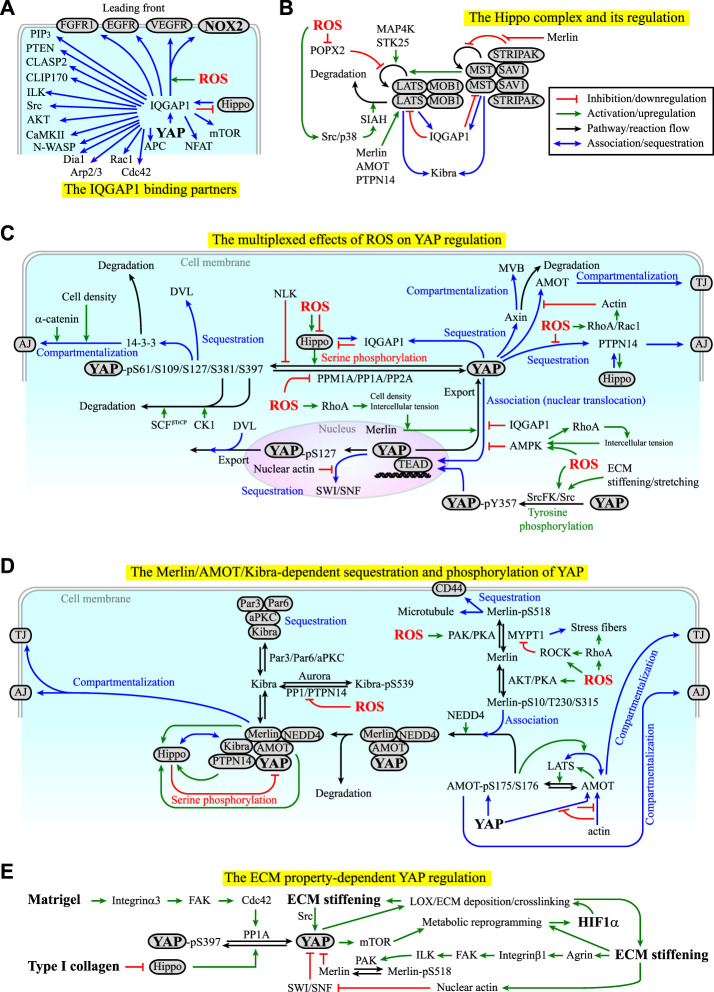
**(A)** Examples of the binding partners of IQ motif-containing GTPase activating protein 1 (IQGAP1). Red lines indicate inhibition or downregulation. Green lines indicate activation or upregulation. Blue lines indicate physical association or recruitment. See Module Boxes IV and V for details. **(B)** The Hippo complex is regulated by itself and several kinases, phosphatases, and molecular scaffolds in a ROS-dependent manner. See Module Box V for details. Black lines indicate the flow of the pathways, cascades, or a reaction. **(C)** ROS exhibit both positive and negative effects on the regulation of YAP signaling manifested in the phosphorylation, dephosphorylation, sequestration, degradation, and compartmentalization of YAP. Red texts indicate inhibition. Blue texts indicate sequestration, association, or compartmentalization. Green texts indicate activation. **(D)** Merlin, angiomotin (AMOT), kidney and brain expressed protein (KIBRA), and protein tyrosine phosphatase non-receptor type 14 (PTPN14) act with the Hippo complex and cytoskeletons to regulate the phosphorylation, sequestration, compartmentalization, and degradation of YAP in a ROS-dependent manner. See Module Box V for details. **(E)** The ECM components and mechanical properties can regulate YAP signaling in a self-perpetuating manner. See Main Text and Module Box V for details.

From the molecular signaling perspective, X-ROS and cell mechanics act both upstream and downstream of each other. This reciprocal coupling occurs through the cytoskeletal dynamics. On the one hand, X-ROS can activate Ras-related C3 botulinum toxin substrate 1 (Rac1) through, for example, the X-ROS-Ca^2+^-PKC coupling (Module Box IV and Figure 3C), and Ras homolog family member A (RhoA), through, for example, cysteine oxidization on the Rho GEF ARHGEF1 (MacKay et al., 2017) (Module Box IV and Figure 3D), by which they promote actin filament polymerization and actomyosin contractility. X-ROS-mediated cysteine oxidization also enables the association of Ras GTPase-activating-like protein or IQ motif-containing GTPase activating protein 1 (IQGAP1) with NOX2 and cytokine receptors such as VEGF receptor (VEGFR) at the lamellipodial leading front of migrating cells (Ikeda et al., 2005; Kaplan et al., 2011) (Figure 4A). IQGAP1 is a scaffold protein that binds to microtubule plus-end binding proteins such as cytoplasmic linker associated protein 2 (CLASP2), YAP, and the regulators of YAP in the Hippo pathway, MST2, and LATS1 (Watanabe et al., 2009; Sayedyahossein et al., 2016; Quinn et al., 2021) (Module Box IV and Figure 4A). As a result, X-ROS signaling influences cell mechanics by modulating cytoskeletal dynamics and the distribution of mechano-transducers such as YAP. On the other hand, actin enhances NOX-mediated ROS production, and an actin-binding site has been identified on the subunit of NOX2, p47^phox^ (Tamura et al., 2006) (Module Box III). p47^phox^ is redox-sensitive, and sequential phosphorylation and S-glutathionylation of p47^phox^ leads to sustained O_2_^−^ production (Nagarkoti et al., 2018). These lines of evidence suggest a self-perpetuating amplification of the ROS-dependent cytokine/ECM signaling and cytoskeletal dynamics.

The effect of ROS on cytoskeletal dynamics appears to depend on the ROS levels. It has been shown that ROS at low (physiologically relevant) levels promote actin filament polymerization, stress fiber assembly, and microtubule self-organization, yet ROS at high levels compromise these processes (Khairallah et al., 2012; Xu et al., 2017; Loehr et al., 2018) (Figure 2D). The explicit mechanisms remain elusive (Wilson and Gonzalez-Billault, 2015). To date, the most well-studied example of the coupling of NOX-ROS and cell mechanics is the microtubule-dependent X-ROS signaling in cardiomyocytes and skeletal muscle cells (Prosser et al., 2011; Prosser et al., 2013; Kerr et al., 2015; Limbu et al., 2015; Robison et al., 2016; Caporizzo et al., 2018; Chen et al., 2018; Caporizzo et al., 2019; Scarborough et al., 2021; Uchida et al., 2021) (Module Box IV). With the interdependence between cytoskeletal dynamics and X-ROS signaling, it is plausible that X-ROS signaling is sensitive to the mechanical modulation in cell morphogenesis and acts in part as a mechano-transducer. The integration of these effects can lead to a self-perpetuated amplification of the cellular mechanical responses, which might serve as a switch for the selection of stem cell fate (see examples in Module Box IV).

### The Coupling of X-ROS-Hypoxia/Cytokine/ECM Signaling With YAP Signaling

One goal of cell/tissue mechanics is to shape organs and tissues into the proper form. In this process, what is needed is the control over the proliferation and differentiation of stem cells and tissue-specific progenitor cells. The fundamental question is how these cells know when and where to stop growing after the organ reaches a certain size and topology. In principle, the growth control should arise from a proper balance of three cellular processes, namely, cell division, cell differentiation, and programmed cell death (apoptosis), in a time- and space-dependent manner. The classical “chemical-driven” view on the control of organ size and topology was started by Alan Turing’s famous work on the dynamic instability of interacting morphogens (Turing, 1952) and is amplified by the focus of molecular biology and genetics on regulatory mechanisms implemented by diffusive molecules. However, attempts to create organ-scale tissues by diffusive morphogens have limited success. Indeed, if organ pattern formation relies on chemical gradients only, it would be impossible to explain several remarkable examples of ordered proliferation, differentiation, and self-organization of the entire organ spontaneously emerging *in vitro* from naive cells cultured in media saturated with mitogens and growth factors (Sasai, 2013). Using soluble factors alone also makes it difficult to realize how fluctuating microenvironments can robustly template cell behavior in time and space with micrometer accuracy (Huang and Ingber, 1999; Discher et al., 2009). It appears that a “mechanics-driven,” non-autonomous effect must exist; in other words, the tissue is endowed with a capacity to inform individual cells with certain “structure-code messengers” about its size and entire topology (Nelson et al., 2006; Piccolo et al., 2014), by which a long-range regulation can be imposed on individual cells (Guo et al., 2012), guiding them to shape the tissue in synchrony with other cells.

The transcriptional coactivators, YAP/TAZ, which boost organ growth and are suppressed by the Hippo complex (Module Box V and Figure 4B), are likely to be the “structure-code messengers” in organ development, homeostasis, repair, and tumorigenesis (Wang et al., 2009; Li et al., 2010; Lian et al., 2010; Zhao et al., 2010a; Dupont et al., 2011; Yu et al., 2015; Panciera et al., 2017). The activity of YAP/TAZ is mainly regulated through PTMs (e.g., serine/threonine and tyrosine phosphorylation and dephosphorylation, and ubiquitination), sequestration, and compartmentalization (Figure 4C). The effectors modulating the PTMs of YAP include the Hippo pathway components such as MST1/2, SAV1, LATS1/2, MOB1, MAP4Ks, and STK25, tyrosine kinases such as Src kinase, the E3 ubiquitin ligase SCF^β-TrCP^, protein phosphatase (PP), and protein tyrosine phosphatase (PTP) (Module Box V). We should point out that the consequences of serine/threonine phosphorylation and tyrosine phosphorylation of YAP are different. While the serine/threonine phosphorylation of YAP promotes YAP sequestration, compartmentalization, or ubiquitination, the tyrosine phosphorylation of YAP promotes YAP nuclear translocation and signaling (Rosenbluh et al., 2012; Smoot et al., 2018; Sugihara et al., 2018) (Figure 4C). For the sequestration of YAP, the major adaptors and scaffold molecules include 14-3-3, α-catenin, Dishevelled (DVL), angiomotin (AMOT), IQGAP1, kidney and brain expressed protein (KIBRA), Merlin, Expanded (Ex), protein tyrosine phosphatase non-receptor type 14 (PTPN14), and Switch/Sucrose non-fermentable (SWI/SNF) (Module Box V and Figures 4C,D). Among them, the association of YAP with AMOT in the cytoplasm is under competition with actin filaments, hence linking cytoskeletal dynamics to YAP regulation (Mana-Capelli et al., 2014) (Module Box V and Figures 4C,D). Likewise, in the nucleus, polymerized nuclear actin filaments (induced by, e.g., the exposure of cells to stiff ECM) bind to SWI/SNF and relieve its sequestration of YAP (Chang et al., 2018). Cell mechanics are also linked to the Merlin-dependent YAP regulation. Merlin phosphorylation at S518, for example, is counteracted by myosin phosphatase target subunit 1- (MYPT1-) regulated PP1c, the phosphatase for myosin light chain (MLC) (Jin et al., 2006; Kiss et al., 2019; Alvarez-Santos et al., 2020). When RhoA, Rho-associated kinase (ROCK), or both are activated (e.g., by integrin–ECM interactions), MYPT1 can be inhibited by ROCK (Kawano et al., 1999) and/or sequestered to stress fibers (Joo and Yamada, 2014), thereby maintaining Merlin at the inactive, S518-phosphorylated state (Module Box V and Figure 4D). The compartmentalization of YAP mainly occurs at adherens junctions (AJs, by, e.g., PTPN14, 14-3-3, and Merlin), tight junctions (TJs, by, e.g., AMOT and Merlin), and multi-vesicular body (MVB, by, e.g., axin) (Module Box V and Figures 4C,D).

Several mechanisms have been identified to activate YAP signaling in an X-ROS- and/or cell mechanics-dependent manner. These mechanisms are to change the PTMs, the sequestration, and/or the compartmentalization states of YAP. Examples of the processes include 1) enhancing the degradation or dephosphorylation of LATS (Kim P. R. et al., 2020; Zhao et al., 2020) (Figure 4B), *2*) reducing YAP S127/S397 phosphorylation (e.g., by PP1A, PP2A, PPM1A (Schlegelmilch et al., 2011; Li et al., 2016; Hu et al., 2017; Zhou et al., 2021), or Nemo-like kinase (NLK) (Moon et al., 2017)) (Figure 4C), *3*) reducing YAP-Merlin association (by, e.g., enhancing Merlin S518 phosphorylation (Morrison et al., 2001; Sherman and Gutmann, 2001)) (Figure 4D), and *4*) attenuating YAP-AMOT association (by, e.g., promoting actin filament polymerization to compete for binding to AMOT (Mana-Capelli et al., 2014)) (Figures 4C,D). A mechanism similar to example 4 is to reduce YAP-SWI/SNF association by nuclear actin filament polymerization (Chang et al., 2018) (Figure 4C). The effects of X-ROS in these processes are complex, as they can be additive, synergistic, or contradicting. To demonstrate such complexity, we use ROS-mediated LATS degradation and dephosphorylation as an example.

The degradation of LATS is primarily mediated by the E3 ubiquitin ligase, SIAH2 (Ma et al., 2015; Zhao et al., 2020) (Figure 4B), the enzyme targeting PHDs for degradation (Nakayama and Ronai, 2004; Qi et al., 2013) (Figure 2C), thus connecting the regulation of hypoxia responses with YAP signaling. SIAH2 can be upregulated by p38 kinase and Src kinase (Khurana et al., 2006; Sarkar et al., 2012), which are redox-sensitive and can be activated by X-ROS (Abe et al., 1996; Aikawa et al., 1997; Kulisz et al., 2002; Emerling et al., 2005; Ray et al., 2012; Xu et al., 2017; Koundouros and Poulogiannis, 2018; Basu et al., 2019; Perillo et al., 2020). This effect places X-ROS upstream of YAP activation (Figure 4B). On the contrary, the dephosphorylation of LATS is primarily mediated by POPX2, which is also redox-sensitive and can be inhibited by ROS through cysteine oxidation (Kim P. R. et al., 2020). This effect places ROS upstream of YAP suppression (Figure 4B). Thus, X-ROS exhibit contradicting effects on YAP regulation (Figure 4C).

Contradicting effects, in fact, appear in many aspects of the ROS-dependent YAP regulation. For example, ROS can activate not only Src and p38 kinases (which activates SIAH), but also Src family kinase (SrcFK) (Tominaga et al., 2000; MacKay et al., 2017) and PKC (Xu et al., 2017) through cysteine oxidation or ROS-Ca^2+^ coupling (Shirai and Saito, 2002) (Figures 3B,C). PKC and SrcFK, in turn, activate Rac1 (Cathcart, 2004; Brown and Griendling, 2009; Gorlach et al., 2015) and Rho guanine nucleotide exchange factor 1 (ARHGEF1) (MacKay et al., 2017) to promote p21-activated protein kinase (PAK) activation and RhoA activation, respectively. The resulting effects include actin filament polymerization (by Rac1 and RhoA), MLC phosphorylation and stress fiber formations (by RhoA) (Tominaga et al., 2000), and MYPT1 inhibition (Kawano et al., 1999) or sequestration to the phosphorylated MLC (by RhoA) (Joo and Yamada, 2014). Among them, actin filaments compete with YAP for the binding of AMOT, thus releasing YAP from the AMOT-mediated sequestration (Figure 4C). PAK catalyzes Merlin S518 phosphorylation (Shaw et al., 2001) to prevent Merlin from binding to YAP (Figure 4D). RhoA-mediated inhibition and sequestration of MYPT1 prevent MYPT1 from dephosphorylating Merlin^pS518^ (Figure 4D). These effects act additively or synergistically to promote YAP signaling. At the same time, RhoA-mediated ROCK activation at the epithelial circumferential actin belt increases intercellular tension and promotes the release of Merlin from AJs to enable Merlin-mediated YAP nuclear export (Furukawa et al., 2017), thereby suppressing YAP signaling (Figure 4C). If not exported, the nuclear YAP requires the binding of TEAD for signaling, which can be disrupted by 5ʹ AMP-activated protein kinase- (AMPK-) mediated YAP phosphorylation at S94 (Mo et al., 2015), and elevated ROS levels were found to increase the AMPK activity (Irrcher et al., 2009) (Figure 4C). In addition, ROS can suppress not only POPX2 (which dephosphorylates LATS), but also PP1 (Santos et al., 2016) and PP2A (Rao and Clayton, 2002; Raman and Pervaiz, 2019), both of which can dephosphorylate YAP to promote YAP signaling (Schlegelmilch et al., 2011; Li et al., 2016) (Figure 4C). These inhibitory effects place ROS upstream of YAP suppression and certainly contradict the aforementioned ROS-mediated YAP activation. Moreover, ROS can activate not only Src, p38, PKC, and SrcFK, but also PKA and AKT, yet the effects of the two kinases on Merlin-YAP association are different or even conflicting (Module Box V and Figure 4D). It is thus likely that the effect of X-ROS on YAP signaling is multiplexed and dependent on the context of the niche and the cellular status.

One consistent influence of X-ROS on YAP signaling is to promote the association of YAP with IQGAP1 (Figure 4C), which brings YAP to the cell leading front (Figure 4A). Another consistency is the effect of intercellular tension on the regulation of YAP signaling. In epithelial organs, the intercellular tension is primarily determined by the contractility at the circumferential actin belt around the AJs. RhoA/ROCK-mediated enhancement of tension at the circumferential actin belt has been shown to promote the release of Merlin from AJs, thereby facilitating Merlin-mediated YAP nuclear export (Furukawa et al., 2017). Consistently applying forces at E-cadherin to mimic the high intercellular tension state has been shown to activate AMPK (Bays et al., 2017), which disrupts the YAP-TEAD association and suppresses the nuclear signaling of YAP (Mo et al., 2015). Moreover, the activated AMPK reinforces the RhoA/ROCK/MLC-mediated contractility to keep the cells at a high-tension state, thereby forming a positive feedback loop for the maintenance of the epithelial barrier (Bays et al., 2017) and the suppression of YAP signaling (Figure 4C). The third consistency is the ROS-mediated activation of tyrosine kinases and suppression of tyrosine phosphates. Unlike the negative regulation of serine phosphorylation of YAP by LATS and other kinases such as AKT and JNK (Basu et al., 2003; Danovi et al., 2008), tyrosine phosphorylation of YAP (at, e.g., Y357) by the redox-sensitive Src kinase or SrcFK promotes the nuclear translocation and signaling of YAP (Rosenbluh et al., 2012; Smoot et al., 2018; Sugihara et al., 2018) (Figure 4C). ROS-activated Src kinase can also suppress LATS by upregulating SIAH2 (Figure 4B), and ROS can inhibit tyrosine phosphates (Hecht and Zick, 1992; Lewis and Aitken, 2001; Chao et al., 2011) such as PTPN14, the inhibition of which abolishes the PTPN14-mediated sequestration of YAP (Liu et al., 2013) (Figure 4C). As a result, the regulations of ROS on tyrosine kinases and phosphatases lead to a synergistic or additive effect on YAP signaling.

X-ROS can be generated in integrin-ECM signaling and cell migration (Module Box III and Figures 3B–D). In these processes, integrin-ECM signaling can promote the dephosphorylation of YAP^pS397^, likely through an integrin α3-FAK-Cdc42-PP1A cascade, leading to the YAP nuclear translocation and potentiating mTOR signaling in stem cell-based tissue renewal (Hu et al., 2017) (Figure 4E). Stiffening and stretching of ECM also leads to Src kinase activation (Koudelkova et al., 2021), which in turn promotes tyrosine phosphorylation and nuclear translocation of YAP (Figures 4D,E). In fact, the mechano-chemical properties of ECM, such as ECM stiffness and ECM components, exhibit a profound impact on YAP signaling. The type I collagen, for example, can stimulate YAP nuclear translocation to suppress adipogenic differentiation in preadipocytes, likely through downregulating the expressions of Hippo pathway kinases (Liu X. et al., 2020). The crosslinking of collagen by, for example, LOX and LOX-like (LOXL) enzymes (Levental et al., 2009) increases ECM stiffness to promote YAP nuclear translocation (Dupont et al., 2011; Noguchi et al., 2018) and metabolic reprogramming (Ge et al., 2021) which can potentially activate HIF-1 signaling (Halligan et al., 2016). HIF-1α signaling and YAP signaling, in turn, can induce the expression of genes responsible for collagen deposition and stiffening directly (Gilkes et al., 2013; Ji et al., 2013) and indirectly (Liu et al., 2015; Noguchi et al., 2017), leading to a self-perpetuating vicious cycle in tissue fibrosis (Noguchi et al., 2018). Another example of the influence of ECM on YAP signaling is Agrin, an ECM proteoglycan that transduces ECM stiffness and cell rigidity to YAP signaling. Agrin activates p21-activated protein kinase- (PAK-) 1 through the integrinβ1-FAK-integrin-linked kinase (ILK) signaling axis, which subsequently phosphorylates Merlin at S518 (Chakraborty et al., 2017) and reduces YAP-Merlin association (Module Box V and Figures 4D,E). Reciprocally, the effect of YAP on ECM remodeling often requires the presence of other niche factors such as TGFβ (Fujii et al., 2012; Noguchi et al., 2018). TGFβ also enhances the association of SIAH2 with LATS2 (Ma et al., 2016) for degradation. These lines of evidence place X-ROS-coupled cytokine/ECM signaling and cell mechanics upstream of YAP signaling. Nevertheless, we should point out that ROS are generally considered an inducer of premature senescence and aging (Kodama et al., 2013; Davalli et al., 2016; Marazita et al., 2016), and YAP signaling can prevent premature senescence yet often lead to tumorigenesis (Xie et al., 2013; Xu X. et al., 2021). How to optimize their interplay to boost longevity while minimizing the risk of tumorigenesis will be an interesting subject to investigate.

YAP signaling dictates the selection of cell fate, and it is likely that YAP signaling follows switch-like behavior. For the therapeutic purpose, it will be convenient if ROS-mediated effects also act as a switch at different stages of stem cell development and tumor progression, whereby pharmaceutical interventions can be explicitly applied to turn “on” or “off” specific or unwanted effects (Kim P. R. et al., 2020). In fact, switch-like enhancement of YAP-mediated epithelial-mesenchymal transition (EMT) has been proposed in cell migration on substrates engineered with nano-scale topographic cues (Park et al., 2019). The potential coexistence of the compatible and conflicting ROS-mediated effects on YAP signaling suggests that X-ROS and cell mechanics regulate YAP activity in a multiplex, niche factor context-dependent manner and can lead to a differential rather than switch-like response. Whether there is segregation between differential and switch-like YAP responses in the variation of niche factors and how such segregation depends on the physiological or pathological niche conditions remain to be resolved.

### The Coupling of X-ROS With HIF/YAP/Notch Triad and PD-L1 Signaling

**FIGURE 5:**
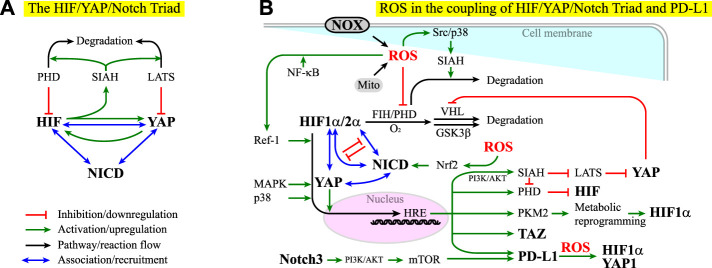
**(A)** HIF, YAP, and Notch act as a triad in that their effectors can associate to influence each other. See Module Box VI for details. NICD stands for the notch intracellular domain. Red lines indicate inhibition or downregulation. Green lines indicate activation or upregulation. Blue lines indicate physical association or recruitment. Black lines indicate the flow of the pathways or a reaction. **(B)** ROS exhibit both positive and negative effects on the coupling of the HIF/YAP/Notch triad and PD-L1 signaling. See the main text and Module Box VI for details.

The involvement of SIAH2 in X-ROS-HIF-1α and X-ROS-YAP signaling suggests that HIF and YAP might be interdependent or connected in the regulation of cell fate and tissue responses. In fact, YAP forms complexes with HIF-1α and acts as the transcription activator of HIF-1α (Xiang et al., 2015; Zhao et al., 2020), and HIF-1α was found to promote YAP activation (Li H. et al., 2018). Positive feedback thus appears in the HIF/SIAH/YAP axis, which might play an important role not only in stem cell physiology but also in tumorigenesis (Module Box VI and Figure 5A). The tumor microenvironment (TME) is often characterized by an abundance of ROS and the stiffening of ECM. From the discussion in the previous sections, we note that both HIF-1α and YAP are sensitive to the ECM stiffness and ROS and that the yield of ROS depends on the O_2_ concentration and the metabolic activities in the TME. An intriguing question is then how the YAP target genes are differentially regulated by ROS-independent and ROS-dependent HIF signaling in response to the change of ECM stiffness and niche O_2_ concentrations. Unfortunately, no quantitative data on this perspective are available to date, and studies are thus warranted.

The complexity in HIF-YAP coupling increases when Notch signaling is considered. In contrast to the regulation of organ size by the Hippo pathway (Yu et al., 2015), Notch signaling regulates the exquisite timing and spatial programming in the organ plan, including the spatiotemporal specification of cell fate and cell differentiation, tissue patterning, and the maintenance of stem cells (Artavanis-Tsakonas et al., 1999; Lasky and Wu, 2005; Sirin and Susztak, 2012; Kessler et al., 2015; Teo et al., 2019). Notch signaling is also associated with a neurological disorder, inflammation, senescence, aging, tumorigenicity, cancer drug resistance, cancer metastasis, cancer stemness, and cancer immune evasion (Sharma et al., 2011; Liu et al., 2012; Wang et al., 2014c; Balistreri et al., 2016; Hoare and Narita, 2018; Wu et al., 2018; Liu et al., 2021a; Xiu et al., 2021). YAP/TAZ forms a complex with the Notch effector, Notch intracellular domain (NICD), to promote the transcription of Notch target genes (Manderfield et al., 2012). Recent studies suggest a coupling of YAP/TAZ and Notch signaling pathways. This coupling can be positive or negative, with YAP/TAZ acting upstream of, downstream of, or in parallel with Notch signaling (Module Box VI). Moreover, YAP/TAZ, HIF-1α, and HIF-2α can bind to NICD to promote the transcriptional activity in a mutually exclusive manner (Hu et al., 2014) (Module Box VI and Figure 5A). Such HIF-Notch coupling can be found in, for example, neurological disorder and degeneration, brain function and angiogenesis, and the maintenance of glioblastoma stem cells (Gustafsson et al., 2005; Chen et al., 2010; Qiang et al., 2012; Hu et al., 2014; Li Y. et al., 2018; Kim S. et al., 2020). Conversely, Notch signaling is required for HIF to preserve the full pluripotency of stem cells under hypoxia (Gustafsson et al., 2005), the condition wherein stem cells maintain their stemness (Ezashi et al., 2005). These lines of evidence suggest that HIF, YAP, and Notch act as a triad in the regulation of stem cell physiology and the dysregulation of cell behavior in tumorigenesis.

In addition to YAP and HIF, recent studies have shown that Notch is associated with various subtypes of X-ROS signaling and involved in oxidative stress (Zhang H.-M. et al., 2018). For example, reciprocal ROS-Notch signaling has been identified in the clusters of circulating tumor cells (CTCs) and myeloid-derived suppressor cells (MDSCs), where CTCs have been considered as the *bona fide* precursors for metastatic tumors and MDSCs, a group of undifferentiated, bone marrow-derived heterogeneous cells with enhanced ability of immune suppression (Gabrilovich and Nagaraj, 2009; Wen et al., 2020), are known to promote neoplastic growth by inhibiting the tumoricidal activity of T cells (Aceto et al., 2014; Boral et al., 2017; Sprouse et al., 2019). Several mechanisms have been identified in X-ROS/cytokine/ECM signaling-coupled Notch signaling. The first is to act through the coupling of TGFβ1 and NOX4-derived ROS in epithelial cells, where niche factor TGFβ1 induces NOX4 expression (through p38 kinase (Ning et al., 2002)), ROS-dependent Nrf2 activation and expression, NOX4-derived ROS production, and Nrf2-dependent Notch signaling (Yazaki et al., 2021), which in turn induces EMT (Matsuno et al., 2012). Herein, Nrf2 stands for nuclear factor erythroid-derived 2-related factor 2, a leucine-zipper transcription factor (Moi et al., 1994). Nrf2 and its repressor Kelch-like ECH-associated protein 1 (Keap1) act as the major regulators for cell redox levels (Sporn and Liby, 2012). It has been shown that elevated ROS levels alone are sufficient to trigger Notch signaling for the homeostasis of airway basal stem cells in an Nrf2-dependent manner (Paul et al., 2014) (Figure 5B). The second is to act through the combination of the GSK3β-mediated crosstalk between Notch and Wnt/β-catenin signaling pathways (Force and Woodgett, 2009; Caliceti et al., 2014), the X-ROS-mediated activation of GSK3β (Wang C.-Y. et al., 2014), and the downregulation of β-catenin by a redox-sensitive negative regulator of Wnt signaling pathway, nucleoredoxin (NRX) (Shin et al., 2004; Funato and Miki, 2010; Funato et al., 2010). Note that GSK3β is also involved in the HIF-α subunit regulation (Flugel et al., 2007) (Module Box I and Figures 2B,C) and the axin-dependent YAP degradation and compartmentalization (Azzolin et al., 2014) (Module Box V and Figure 4C). The third is to act through niche mechanics- and ROS-interdependent integrin signaling (Werner and Werb, 2002; Gregg et al., 2004; Buricchi et al., 2007; Taddei et al., 2007; Zeller et al., 2013; Xu Z. et al., 2021), where the activation of ILK potentiates Notch signaling (Maydan et al., 2010) and regulates GSK3β activity (Maydan et al., 2010).

YAP signaling can upregulate PD-L1, the ligand for the cell surface glycoprotein PD-1 that suppresses immune responses in chronic inflammation and in the tumor microenvironment (TME) (Greenwald et al., 2005; Janse van Rensburg et al., 2018), particularly in cancer cells (Lee et al., 2017; Miao et al., 2017). However, YAP is not alone. Recent studies have identified a Notch signaling pathway through the Notch3-PI3K-AKT-mTOR axis to be responsible for the overexpression of PD-L1 in breast cancer stem cell-like (CSC-like) cells (Mansour et al., 2020) (Figure 5B). Under hypoxia, the common niche condition in the TME, HIF-1α but not HIF-2α, has been found to bind to an HRE in the PD-L1 promoter region to overexpress PD-L1 in myeloid-derived suppressor cells (MDSCs) (Noman et al., 2014), by which HCCs evade immune systems (Wen et al., 2020). A concomitant elevation of cell surface PD-L1 and intracellular HIF-2α expression has also been observed in solid tumors (Chang et al., 2016; Tawadros and Khalafalla, 2018; Guo et al., 2019; Zhou et al., 2019), where enhanced activities in ERK, AKT, IκBα (nuclear factor of kappa light polypeptide gene enhancer in B-cells inhibitor, alpha), and NF-κB were found to be involved in PD-L1 overexpression (Guo et al., 2019). Conversely, PD-L1 overexpression can promote the expression of HIF-1α and YAP-1 in a ROS-dependent manner (Tung et al., 2018), perhaps through the interaction of PD-L1 with vimentin, a major cytoskeletal element contributing to cell stiffness and EMT (Ancel et al., 2019), or through the nuclear translocation of PD-L1 and subsequent operation on a panel of immune regulation-related genes (Gao et al., 2020; Jaccard and Ho, 2020) (Figure 5B).

The coupling of PD-L1 and HIF/YAP/Notch signaling has led to a proposed idea that the targeting therapy on HIF/YAP/Notch signaling pathways, along with the conventional chemotherapy and immune therapy, might serve as a potential surrogate for cancer treatment (Janghorban et al., 2018) (Module Box VI). Given the coupling of X-ROS in HIF/YAP/Notch signaling, it is legitimate to ask whether niche ROS affect PD-L1 expression and/or signaling. Figure 2E shows that when the yield of free electrons from the respiratory chain (i.e., ETC) exceeds a certain level with respect to the niche oxygen concentration, ROS can be created and leak to the cytoplasm. This situation is likely to occur at the TME, where tumor cells often carry enhanced glycolysis. In addition, the TME contains inflammatory cells that produce a significant amount of ROS through, for example, NOX, and modify the oxidative stress of the TME, which in turn can influence the antitumor effect of immune responses. It is, therefore, important to evaluate the impact of ROS on PD-L1 expression and functions (Bailly, 2020). This impact is complex and bi-directional. For example, X-ROS and cell mechanics can upregulate HIF and YAP signaling activities and expression levels (Abe et al., 1996; Aikawa et al., 1997; Kulisz et al., 2002; Emerling et al., 2005; Ray et al., 2012; Hu et al., 2017; Xu et al., 2017; Koundouros and Poulogiannis, 2018; Basu et al., 2019; Perillo et al., 2020), which in turn promote PD-L1 expression (Noman et al., 2014; Janse van Rensburg et al., 2018). Conversely, PD-L1 can induce HIF-1α expression in a ROS-dependent manner and, in turn, upregulate YAP1 expression (Tung et al., 2018) (Figure 5B). These lines of evidence suggest a potential self-perpetuating amplification in the ROS-HIF/YAP-PD-L1 axis. As a result, enhancing ROS production might promote the PD-L1 expression, and scavenging ROS can repress the PD-L1 expression. Nevertheless, there are contradicting examples in cancer cell lines, where applying ROS-generation drugs leads to PD-L1 downregulation and applying ROS scavengers promotes PD-L1 expression (Bailly, 2020). More studies on the interplay of ROS and PD-L1 are thus warranted.

## Conclusion Remarks

Except for the anti-pathogen capacity, ROS have long been considered harmful due to the ability to damage DNA and proteins but is now recognized as an important element in regulating stem cell physiology. Exploding evidence over the past decade further indicates that ROS are intensively coupled with tissue mechanics and HIF-YAP-Notch signaling. Such coupling is manifested in organ development, homeostasis, and repair, and when things go wrong, the coupling can lead to tumorigenesis. This review discusses the interplay of ROS (particularly NOX-derived ROS (i.e., X-ROS)) and the HIF-YAP-Notch signaling. The potentiation of PD-L1 expression in response to ROS-HIF-YAP-Notch signaling is also addressed. Most importantly, we point out the existence of multiplexed positive and negative feedback couplings that occur at different times (i.e., transient or prolonged) and spatial (i.e., autonomous (within the cell) or non-autonomous (within the niche)) scales. Understanding under what niche conditions these couplings can lead to differential or switch-like tissue responses and/or change self-sustained regulation in stem cell physiology to self-perpetuating dysregulation in cancer progression will help us move into the clinical realm to design strategies for stem cell-based and X-ROS-targeting therapy.

## Supporting Boxes

### Math Box I: The Estimated Phase Diagram of ROS Production

ROS are mainly produced by mitochondria (Murphy, 2009; Juan et al., 2021). In the regular energy production process, the decomposition of carbon hydrates yields CO_2_ and H_2_, the latter of which forms the high-energy electron donors: nicotinamide adenine dinucleotide phosphate- (NADP-) H, and flavin adenine dinucleotide- (FAD-) H_2_. These donors bring electrons to the mitochondria’s inner membrane electron transport chain (ETC), through which the electrons are delivered to the molecular oxygen O_2_ in exchange for a buildup of pH gradient and an electrochemical potential across the membrane. When the proton flows back through the membrane, it drives the rotation of the membrane-bound ATP synthase and phosphorylates ADP into ATP. This process is called “chemiosmosis,” a process by which oxidative phosphorylation generates ATP (Anraku, 1988; Kracke et al., 2015). Eukaryote ETC consists of NADH-coenzyme Q oxidoreductase (Complex I), succinate-Q oxidoreductase (Complex II), electron transfer flavoprotein-Q oxidoreductase, Q-cytochrome c oxidoreductase (Complex III), and cytochrome c oxidase (Complex IV) (Kracke et al., 2015). Among them, Complexes I, III, and IV are transmembrane proteins coupling the transfer of electrons with the transport of protons. Q stands for ubiquinone, a lipid-soluble electron carrier, and cytochrome c is a water-soluble electron carrier. For an effective electron transfer, the electron donated from NADPH and FADH_2_ should be transported between the lipid-soluble and water-soluble carriers along the membrane to reach the final target Complex IV, where it binds to O_2_ to form H_2_O. In reality, however, the anionic nature of the free electron allows it to escape through the transmembrane complexes to both sides of the inner mitochondrial membrane (Murphy, 2009), where it binds to O_2_ delivered by cytoplasmic oxygen carriers such as cytoglobin (Novianti et al., 2020). This “leakage” primarily occurs at Complexes I/III and, in turn, forms superoxide, O_2_• (or O_2_^−^), a major form of ROS (Murphy, 2009; Bleier and Drose, 2013).

The theoretical value for the reduction of O_2_ to O_2_^−^ in mitochondria was estimated as −68 to −230 mV/mole (Murphy, 2009) and thus is thermodynamically favorable (Andreyev et al., 2005). To see how the free electron selects the “leakage” over the regular path to reach O_2_, we considered the internal electron transfer in the catalytic cycle of Complex IV, which has been documented as the rate-limiting step (Sarti et al., 1988). Complex IV contains four electron carriers, including two heme groups, heme “a” and heme “a3,” each of which contains an iron ion, and two Cu groups, the first of which contains two copper ions and is referred to as CuA/CuA and the second is formed by a single copper ion and referred to as CuB (Voet and Voet, 2011). Complex IV receives free electrons from the water-soluble carrier, cytochrome c, and passes the electrons internally through CuA/CuA to “a,” “a3,” and finally CuB. While the function of CuA/CuA and “a” is primarily for electron transfer, “a3” and CuB form a binuclear center not only for electron transfer but also for O_2_ association and reduction. Adjacent to the binuclear center is a tyrosine group (Tyr244-OH) which also participates in the process of O_2_ reduction. To proceed, we hereafter used the label “***X***” to represent the binuclear center, a3(Fe)-(CuB)-(Tyr244-OH). Likewise, we used “***c***” to denote cytochrome c. In terms of the redox state, we used “***c*** ^0^” and “***c*** ^−^” to indicate the reference state and the reduced state (i.e., carrying one free electron) of cytochrome c, respectively. As for ***X***, its catalytic cycle starts from the reference state, a3(Fe^3+^OH^−^)-(CuB^2+^)-(Tyr244-O^−^) (referred to as ***X*** ^0^). In each cycle, four electrons from four reduced cytochrome c molecules are used, along with the consumption of four protons from the mitochondrial matrix (equivalent to pumping four protons to the intermembrane space). The first electron and proton reduce the copper ion and restore the tyrosine group of ***X*** into a3(Fe^3+^OH^−^)-(CuB^+^)-(Tyr244-OH) (referred to as ***X*** ^−^). The second electron and proton reduce the Fe^3+^ of ***X*** into a3(Fe^2+^)-(CuB^+^)-(Tyr244-OH) (referred to as ***X*** ^2−^), during which the hydroxide ligand, OH^−^, at “a3” is protonated and lost as water, creating a void for O_2_ association. Upon association, the oxygen is rapidly reduced by two electrons from a3(Fe^2+^), one electron from (CuB^+^), and one electron and a proton from (Tyr244-OH). The reduction of O_2_, in turn, transforms ***X*** into the fully oxidized state, a3(Fe^4+^O^2−^)-(CuB^2+^OH^−^)-(Tyr244-O^*^) (referred to as ***X*** ^2+^), where Tyr244-O^*^ indicates a neutral tyrosine radical. Following O_2_ reduction is the addition of the third electron and proton that reduces tyrosine radical and converts ***X*** to a partially oxidized state, a3(Fe^4+^O^2−^)-(CuB^2+^)-(Tyr244-O^−^) (referred to as ***X*** ^+^), with the yield of one water molecule. The fourth electron reduces the iron ion, and with the oxygen atom picking up a proton from the matrix, converts ***X*** back to a3(Fe^3+^OH^−^)-(CuB^2+^)-(Tyr244-O^−^), that is, the ***X*** ^0^ state (Voet and Voet, 2011; Wikstrom and Springett, 2020) (Figure 1B).

In the absence of protein degradation and synthesis, we set (***c*** ^0^ + ***c*** ^−^) = *ρ*_*c*_ and (***X*** ^0^ + ***X*** ^−^ + ***X*** ^2−^ + ***X*** ^+^ + ***X*** ^2+^) = *ρ*_*IV*_, with *ρ*_*c*_ and *ρ*_*IV*_ as the densities of cytochrome c and Complex IV on the mitochondrial inner membrane, respectively. Ignoring the spatial inhomogeneity and fluctuation of free electrons and O_2_, we used the following equations to address the dynamics of *X* and *c*:

$$\frac{dX^{0}}{dt}=k_{IET}c^{-}\left[{\text{H}}^{+}\right]X^{+}-k_{IET}c^{-}\left[{\text{H}}^{+}\right]X^{0},$$ (1)

$$\frac{dX^{-}}{dt}=k_{IET}c^{-}\left[{\text{H}}^{+}\right]X^{0}-k_{IET}c^{-}\left[{\text{H}}^{+}\right]X^{-},$$ (2)

$$\frac{dX^{2-}}{dt}=k_{IET}c^{-}\left[{\text{H}}^{+}\right]X^{-}-k_{O2}{\left[{\text{O}}_{2}\right]}_{m}X^{2-},$$ (3)

$$\frac{dX^{2+}}{dt}=k_{O2}{\left[{\text{O}}_{2}\right]}_{m}X^{2-}-k_{IET}c^{-}\left[{\text{H}}^{+}\right]X^{2+},$$ (4)

$$\frac{dX^{+}}{dt}=k_{IET}c^{-}\left[{\text{H}}^{+}\right]X^{2+}-k_{IET}c^{-}\left[{\text{H}}^{+}\right]X^{+},$$ (5)

$$\frac{dc^{-}}{dt}=k_{ETC}\left[e^{-}\right]c^{0}-k_{IET}\left[{\text{H}}^{+}\right]\left(X^{+}+X^{0}+X^{2+}+X^{-}\right)c^{-},$$ (6)

$$\frac{d\left[e^{-}\right]}{dt}=Y-\left(k_{leak}{\left[{\text{O}}_{2}\right]}_{c}+k_{ETC}c^{0}\right)\left[e^{-}\right].$$ (7)

*k*_*IET*_ was referred to as the internal electron transfer rate from cytochrome c to the binuclear center of Complex IV (for simplicity, we used a single entity to represent all the transfer events). [H^+^] was the proton concentration in the mitochondrial matrix. [O_2_]_*m*_ indicated the mitochondrial molecular oxygen concentration, and *k*_*O*2_ was the association rate with Complex IV. [*e* ^−^] stood for the density of free electron that was generated at a rate ***Y*** and transferred through ETC to the cytochrome c at a rate *k*_*ETC*_, or leaked at a rate *k*_*leak*_ to cytoplasmic O_2_, the concentration of which was set as [O_2_]_*c*_. These parameters and variables were tissue context- and physiology-dependent, and estimates had been made in = previous studies (Murphy, 2009; Wikstrom and Springett, 2020). In principle, [O_2_]_*m*_ could be related to [O_2_]_*c*_. Using an estimate of [O_2_]_*c*_ as 120 μM (Wikstrom and Springett, 2020) and [O_2_]_*m*_ as 25 μM (Murphy, 2009), we could set them at a ratio of ∼0.2.

At the steady state, all of the “*X*” states in Eqs 1–5 could be solved in terms of ***X*** ^0^ and used to express the steady-state solutions of *c* ^−^ and *e* ^−^ in Eqs 6, 7:

$$X^{0}=\rho_{IV}/\left(4+\frac{k_{IET}\left[{\text{H}}^{+}\right]c^{-}}{k_{O2}{\left[{\text{O}}_{2}\right]}_{m}}\right),$$ (8)

$$c^{-}=\rho_{C}k_{ETC}\left[e^{-}\right]/\left(4k_{IET}\left[{\text{H}}^{+}\right]X^{0}+k_{ETC}\left[e^{-}\right]\right),$$ (9)

$$\left[e^{-}\right]=Y/\left(k_{leak}{\left[{\text{O}}_{2}\right]}_{c}+k_{ETC}\left(\rho_{C}-c^{-}\right)\right),$$ (10)

$$z\equiv \frac{c^{-}}{\rho_{C}}=\frac{1}{1+\frac{4\frac{\rho_{IV}}{Y}k_{IET}\left[{\text{H}}^{+}\right]\left(\frac{k_{leak}{\left[{\text{O}}_{2}\right]}_{c}}{k_{ETC}}+\rho_{C}\left(1-z\right)\right)}{4+\frac{k_{IET}\left[{\text{H}}^{+}\right]\rho_{C}}{k_{O2}{\left[{\text{O}}_{2}\right]}_{m}}\times z}},$$ (11)

$$w\equiv \frac{k_{ETC}\left(\rho_{C}-c^{-}\right)}{k_{leak}{\left[{\text{O}}_{2}\right]}_{c}}=\frac{k_{ETC}\rho_{C}\left(1-z\right)}{k_{leak}{\left[{\text{O}}_{2}\right]}_{c}}.$$ (12)

Combining Eqs 8–10, we had Eq. 11, which defined the fraction of reduced cytochrome c with respect to all the cytochrome c on the membrane as *z* (0 ≤ *z* ≤ 1). Examining the left-hand and the right-hand sides of Eq. 11, we found that there was always a solution of *z* between zero and one. In Eq. 12, we defined the ratio of electrons selecting the regular path over the leakage to reach O_2_. When *w* was less than one, most electrons selected the leakage. The critical z* at *w* = 1 was found in Eq. 12:

$$z^{*}=1-\frac{k_{leak}{\left[{\text{O}}_{2}\right]}_{c}}{k_{ETC}\rho_{C}}.$$ (13)

From Eq. 13, the maximal [O_2_]_*c*_ *∗* for *w* ≥ 1 read as follows:

$${\left[{\text{O}}_{2}\right]}_{c}^{*}=\frac{k_{ETC}\rho_{C}}{k_{leak}}.$$ (14)

Eq. 14 suggests a critical cytoplasmic oxygen concentration [O_2_]_c_*, which increases with the density of available cytochrome c on the mitochondrial membrane, *ρ*_*C*_. For cytoplasmic oxygen concentration above [O_2_]_c_*, electrons generated in ETC predominantly leaked and formed ROS. Using Eqs. 11–14, for a given [O_2_]_*c*_, we obtained the critical electron generation rate Y* in the ETC, and for ***Y*** > Y*, the generated electrons predominantly selected the leakage over the regular path to reach O_2_:

$$Y^{*}=\frac{8\rho_{IV}k_{IET}\left[{\text{H}}^{+}\right]\rho_{C}\left(1-\frac{k_{leak}{\left[{\text{O}}_{2}\right]}_{c}}{k_{ETC}\rho_{C}}\right)}{4+\frac{k_{IET}\left[{\text{H}}^{+}\right]\rho_{C}}{k_{O2}{\left[{\text{O}}_{2}\right]}_{m}}\left(1-\frac{k_{leak}{\left[{\text{O}}_{2}\right]}_{c}}{k_{ETC}\rho_{C}}\right)}=\frac{\frac{8\rho_{IV}k_{IET}k_{leak}\left[{\text{H}}^{+}\right]}{k_{ETC}}{\left[{\text{O}}_{2}\right]}_{c}^{*}\left(1-\frac{{\left[{\text{O}}_{2}\right]}_{c}}{{\left[{\text{O}}_{2}\right]}_{c}^{*}}\right)}{4+\frac{k_{IET}k_{leak}\left[{\text{H}}^{+}\right]}{k_{O2}k_{ETC}\frac{{\left[{\text{O}}_{2}\right]}_{m}}{{\left[{\text{O}}_{2}\right]}_{c}}}\times \frac{{\left[{\text{O}}_{2}\right]}_{c}^{*}}{{\left[{\text{O}}_{2}\right]}_{c}}\left(1-\frac{{\left[{\text{O}}_{2}\right]}_{c}}{{\left[{\text{O}}_{2}\right]}_{c}^{*}}\right)}\text{for }\left[\text{O2}\right]\text{c ≤ }\left[\text{O2}\right]*=\mathrm{kETC\rho C/kleak,}Y^{*}=0.$$ (15)

Using the estimate that [O_2_]_*m*_/[O_2_]_*c*_ ∼ 0.2, we obtained the maximal electron generation rate on the variation of cytoplasmic oxygen concentrations (Figure 2E). Below this rate, over 50% of electrons would be used for oxidative phosphorylation.

### Module Box I: O_2_-Dependent Regulation of HIF-α Stability

The cells use the oxygen-sensing regulations to regulate the stability of HIF-α subunits in response to niche oxygen. For HIF-1α, these regulations occur at its functional motifs: proline 402 and 564 at its N-terminal activation domain (NAD) and asparagine 803 at its C-terminal transactivation domain (CTAD). Both NAD and CTAD can recruit E1A binding protein p300 (p300)/cyclic adenosine monophosphate response element-binding protein-binding protein (CREB-binding protein, CREBBP, or CBP) co-activators to enhance the transcriptional activity of HIF. The HIF prolyl hydroxylase domain-containing proteins (PHD or HIF prolyl hydroxylases (HPH)) 1-3 (Fong and Takeda, 2008) and the factor inhibiting HIF (FIH) (Lando et al., 2002; Sim et al., 2018) are the main enzymes responsible for the oxygen-sensing post-translational modifications (PTMs) of HIF-α subunits (Figure 2B). PHD is a Fe^2+^-dependent dioxygenase. It binds to one O_2_ and one HIF-α subunit at the same time, followed by transferring O_2_ to the proline 402 and 564 of the HIF-1α subunit (or 405 and 531 of the HIF-2α subunit) (Hashimoto and Shibasaki, 2015), HIF-L-proline + 2-oxoglutarate (α-ketoglutarate) + O_2_ ⟶ HIF-trans-4-hydroxy-L-proline + succinate + CO_2_, to hydroxylate the proline residues. The reaction indicates that the accumulation of 2-oxoglutarate (or α-ketoglutarate) promotes the hydroxylation of HIF, and the accumulation of succinate prohibits hydroxylation. Once hydroxylated, the proline residue not only fails to recruit p300/CBP to NAD but also becomes recognizable by von Hippel–Lindau tumor suppressor protein (VHL) E3 ubiquitin ligase, which targets the HIF-α subunit for ubiquitination and a rapid 26S proteasome-dependent degradation (Maxwell et al., 1999; Bruick and McKnight, 2001; Epstein et al., 2001; Ivan et al., 2001; Jaakkola et al., 2001). Consequently, PHDs serve as an oxygen sensor to regulate HIF-α subunit stability in response to the fluctuation of niche oxygen concentration [O_2_] (Ivan et al., 2001). In comparison, FIH is an asparaginyl hydroxylase that uses α-ketoglutarate and O_2_ to hydroxylate asparagine 803 of the HIF-1α (or 851 of the HIF-2α) (Schofield and Ratcliffe, 2004) and inhibits its transcriptional activity at CTAD (Lando et al., 2002; Sim et al., 2018), rather than degradation, a detailed discussion of which A detailed can be found elsewhere (Masson and Ratcliffe, 2014; Strowitzki et al., 2019). Apart from hydroxylation, the stability and the ability of HIF-1α to translocate into the nucleus depends on phosphorylation, which is primarily mediated by kinases such as glycogen synthase kinase 3β (GSK3β) and MAPKs (the common effectors involved in TGFβ and IGF-1 signaling, for example, extracellular regulated kinases (Erk1/2) and p38 kinase (Richard et al., 1999; Sodhi et al., 2000; Flugel et al., 2007)) (Figure 2C). As HIF-1α promotes angiogenesis and glycolysis and HIF-2α helps the maintenance of stemness (Figure 2A), it is not surprising that HIFs are dysregulated in tumors. In fact, not only HIF but also PHDs are dysregulated in tumors. PHDs are often overexpressed in tumors by contradicting the expectations, and inhibition of PHDs can impair tumor growth, metastasis, and immune tolerance (Gaete et al., 2021). Thus, HIFs and PHDs have been proposed as therapeutic targets against cancer.

The regulation of HIF stability by FIH and PHD depends on their *K*_m_ values for [O_2_] association. The *K*_m_ value of FIH is ∼90–200 μM (Koivunen et al., 2004; Tarhonskaya et al., 2015). In comparison, the *K*_m_ value of PHD for [O_2_] association is documented as 230–250 μM (Fong and Takeda, 2008) or even higher (250 μM–1.7 mM) (Ehrismann et al., 2007; Dao et al., 2009; Tarhonskaya et al., 2015). In any case, it is above the [O_2_] in air-saturated aqueous buffer at 37°C ([O_2_] ∼21% (∼210 μM)) (Reynafarje et al., 1985; Murphy, 2009), abundantly above the physiological oxygen concentration ([O_2_] ∼7% (∼70 μM)) (Hu et al., 2014), larger than the *K*_m_ value of other oxygenases such as collagen PHD (∼40 μM) (Hirsila et al., 2003), and far above the oxygen concentration in mitochondria ([O_2_] ∼3–30 μM) (Turrens, 2003). Such discrepancy reflects that FIH and PHD are designed for different purposes in response to the niche oxygen concentrations (i.e., FIH for differential activation of HIF target genes and PHD for degradation) and suggests that additional mechanisms might exist for the regulation of HIF-α subunits. Indeed, negative feedback mechanisms have been identified. HIF-1α, for example, promotes its degradation by inducing the expression of PHD2-3 and FIH-1 (Marxsen et al., 2004; Kobayashi et al., 2021).

### Module Box II: ROS-Dependent Regulation of HIF-α Stability

Recent evidence suggests that ROS contribute to the regulation of HIF-α stability by modulating the activity of PHD (Gerald et al., 2004; Fong and Takeda, 2008; Lee et al., 2016) (Figure 2C). The precise mechanism by which ROS regulate PHD is complex and not fully understood. At least three inhibitory and one enhancing mechanisms have been identified. The first inhibitory mechanism is to act by oxidizing the cysteine residues of PHD into disulfide bonds, which cause homo-dimerization and inactivation of PHD (Lee et al., 2016). The second is to act through chelating and oxidizing PHD-bound Fe^2+^ to Fe^3+^, by which the ability of PHD to bind to O_2_ is abolished (Gerald et al., 2004; Fong and Takeda, 2008). The third is to act through seven in absentia homolog 2 (SIAH2), a RING finger-containing E3 ubiquitin ligase targeting PHDs for ubiquitin-mediated proteasome degradation (Nakayama and Ronai, 2004; Qi et al., 2013). SIAH2 can be phosphorylated and activated by several ROS-activated kinases, a detailed review of which can be found elsewhere (Xu and Li, 2021). In contrast, the enhancing mechanism is a long-term effect and acts through redox factor-1 (Ref-1). Prolonged ROS exposure induces Ref-1 expression in an NF-κB- (nuclear factor kappa-light-chain-enhancer of activated B cells-) dependent manner and, in turn, upregulates the transcriptional activity of HIF-1α to promote PHD2 and FIH-1 expressions (Kobayashi et al., 2021), the outcome of which is to downregulate HIF-1α (Figure 2C).

### Module Box III: The NOX-Derived ROS (X-ROS) Signaling

NOX can be found on the plasma membrane (NOX1-5 and Duox1-2), endoplasmic reticulum (NOX2, NOX4, and NOX5), mitochondrial membrane (NOX4), nuclear membrane (NOX4-5), membrane microdomains such as caveolae and lipid rafts (NOX1), focal adhesions (NOX4), and invadopodia (NOX1 and NOX4) (Brown and Griendling, 2009; Brandes et al., 2014a; b; Fukai and Ushio-Fukai, 2020). The catalytic domains of NOX, homologs of gp91^phox^ β subunit with six transmembrane helices, are anchored to the membrane with the cytoplasmic tails binding to NADPH for electron transfer (Brandes et al., 2014b). Upon activation, the NOX-associated NADPH is oxidized, and electrons are transferred across the gp91^phox^ transmembrane domain to bind to O_2_ in the extracellular or intracellular spaces, thereby increasing ROS levels in the niche or inside the cell (Brandes et al., 2014b).

In general, the functionality of NOX requires their catalytic units, the transmembrane gp91^phox^ homolog subunit (each NOX subtype has its own gp91^phox^ homolog), to be in a homodimer (e.g., NOX5) or in a complex with specific membrane scaffolds, namely, NOX1-4 with the membrane scaffold p22^phox^ and Duox1-2 with membrane scaffolds DuoxA1-2, respectively (Brandes et al., 2014b; Skonieczna et al., 2017) (Figure 3A). The mechanisms by which NOX are activated vary among the subtypes. NOX5 and Duox1-2, for example, are activated by calcium binding to their cytoplasmic EF-hand calcium-binding motifs, whereas the activation of NOX1-3 requires the assembly and the PTMs of their cytoplasmic regulators (Brown and Griendling, 2009; Brandes et al., 2014b; Skonieczna et al., 2017). By contrast, NOX4 is constitutively active and does not need the association of any cytoplasmic regulators to produce ROS (Ellmark et al., 2005). Still, the activity of NOX4 is modulated by the phosphorylation on its tyrosine reside 491 (in, e.g., IGF-1 stimulation (Xi et al., 2013)) and on the threonine residues of its membrane scaffold p22^phox^ (Regier et al., 1999). Below, we briefly review the regulation of NOX 1-4 and discuss how they are coupled with cytokine and ECM signaling.

As the first example, we use NOX2 to illustrate how the NOX multi-unit assembly is organized and how the PTMs of NOX subunits affect their assembly and functions. The details can be found elsewhere (Brandes et al., 2014b; Rastogi et al., 2016; Skonieczna et al., 2017). As aforementioned, NOX1-3 are inactive when present as a monomer (i.e., with the gp91^phox^ homolog subunit alone) and need to form a complex with the membrane scaffold p22^phox^ for maturation and stabilization (Nakano et al., 2007). The activation of NOX2 further requires recruiting p21^Rac1/2^ (Rho GTPase Rac1/2), p67^phox^, p40^phox^, and p47^phox^ into the gp91^phox^-p22^phox^ complex (Brandes et al., 2014b), where gp91^phox^ is the core subunit and each NOX subtype has its own homolog (Figure 3C). Recruiting these molecules consumes high-energy phosphate compounds. For example, the recruitment of Rac needs the Rac guanine nucleotide exchange factor (GEF) to switch Rac from the GDP- to GTP-bound form and expose its prenylated tail for membrane binding (Abo et al., 1994; Diekmann et al., 1994), whereas the recruitment of p47^phox^ requires phosphorylation of p47^phox^ by serine/threonine kinases such as protein kinase B (PKB)/AKT, protein kinase C (PKC), and p21-activated protein kinase (PAK) (Fontayne et al., 2002; Chen et al., 2003; Hoyal et al., 2003; Bey et al., 2004; Martyn et al., 2005). Reciprocally, the association between p22^phox^ and p47^phox^ is enhanced if p22^phox^ is phosphorylated by phosphatidic acid- (PA-) activated protein kinase or PKC (Regier et al., 1999). The phosphorylation of p47^phox^ not only enables its binding to p67^phox^ and p22^phox^ but also exposes its pbox consensus sequence (PX) domain to phosphatidylinositol (4,5)-bisphosphate or phosphatidylinositol (3,4,5)-trisphosphate (PIP_2_ or PIP_3_) for membrane binding (Ago et al., 2003; Groemping et al., 2003). Such PX domain is also found in p40^phox^, a regulator that binds to p67^phox^ and stabilizes p67^phox^-p47^phox^ complex formation (Kanai et al., 2001).

The involvement of PIP_2_/PIP_3_ in NOX2 multi-unit assembly suggests that NOX2 activity is modulated by PI3K and phosphatase and tensin homolog (PTEN), the common effectors in the cytokine/ECM signaling. Likewise, the involvement of phosphorylation-mediated PTMs on the subunit assembly suggests that cytokine/ECM signaling regulates NOX2 activity. These phosphorylation-mediated PTMs are not just for potentiating NOX multi-unit assembly. PKC-mediated phosphorylation in NOX2 and p67^phox^, for example, has been found to maximize the yield of NOX2-derived ROS (Regier et al., 1999; Raad et al., 2009; Brandes et al., 2014b), which in turn can evoke calcium influx (Gorlach et al., 2015). The binding of Ca^2+^ to the C2 domain of PKC then promotes the membrane targeting of PKC (Shirai and Saito, 2002) and the phosphorylation of p47^phox^, p67^phox^, p40^phox^, and Rac through calcium-activated PKC (Cathcart, 2004; Brown and Griendling, 2009; Gorlach et al., 2015; Islam et al., 2018; Tu et al., 2020), leading to a potential feedback amplification along the ROS-Ca^2+^-PKC signaling axis (Figure 3C).

The second example is NOX1 and NOX3, the activation of which requires the assembly of p47^phox^ homolog Noxo1 and the p67^phox^ homolog Noxa1 to NOX1-p22^phox^ and NOX3-p22^phox^ complexes, respectively (Brandes et al., 2014b). Similar to p67^phox^, the activity of Noxa1 is regulated by phosphorylation. Unlike p67^phox^, however, the phosphorylation of Noxa1 can lead to active or inhibitory effects, which involve not only serine/threonine kinases but also protein tyrosine kinases. For example, PKC, steroid receptor coactivator (Src) kinase, and CaMK2 phosphorylate Noxa1 and enhance its association with Noxo1 and NOX1, whereas the phosphorylation of Noxa1 by cAMP-dependent protein kinase or PKA inhibits the association (Kim et al., 2007; Gianni et al., 2010; Kroviarski et al., 2010; Brandes et al., 2014b). Inhibitory phosphorylation also occurs at NOX2 (mediated by casein kinase 2 (CK2)) (Kim et al., 2009) and at p40^phox^, the phosphorylation of which leads to the suppression of NOX activity (Lopes et al., 2004).

The third example is NOX4 (Figure 3D). The activation of NOX4 does not explicitly require the multi-unit assembly of cytosolic regulators. Still, NOX4 interacts with several cytosolic molecules to modulate its activity. For example, NOX4 interacts with a chaperon protein, calnexin, to facilitate its maturation (Prior et al., 2016). NOX4 also interacts with a mitochondrial protein, polymerase δ-interacting protein 2 (Poldip2), to increase its activity (Lyle et al., 2009). Poldip2 is a molecule interacting with DNA polymerase δ p50 subunit and with the proteins constituting the mitochondrial DNA nucleoid, through which NOX4 activity is associated with the TCA cycle and metabolic reprogramming (Andjongo et al., 2021; Kulik et al., 2021). In fact, metabolism-related cytokines, such as insulin and IGF-1, are known to increase NOX4 expression (Meng et al., 2008; Schroder et al., 2009; Kim et al., 2012). Cytokine-enhanced upregulation of NOX has also been reported in the TGFβ-mediated pulmonary remodeling (Watson et al., 2016) (Figure 3B). In addition to the mitochondria, Poldip2 interacts and activates the Rho GEF, epithelial cell transforming sequence 2 (Ect2), to enhance actin filament polymerization, thereby linking the NOX4 activity to cytoskeletal dynamics (Huff et al., 2019). As for the phosphorylation-mediated PTMs, in contrast to NOX1-3, mostly regulated by serine/threonine kinases, NOX4 is primarily regulated by protein tyrosine kinases (PTKs) such as Src kinase. The phosphorylation of Tyr-491 on NOX4, for example, promotes NOX4 association with Src homology 2- (SH2-) domain-containing protein tyrosine phosphatase (SHP) substrate-1 (SHPS-1), through an adaptor protein, growth factor receptor-bound protein 2 (Grb2) (Xi et al., 2013). SHPS-1 is a transmembrane protein that serves as a scaffold to cluster membrane receptors such as IGF-1 receptor (IGF-1R) with other signaling and adapter molecules, including protein tyrosine kinases, Src family kinase (SrcFK), focal adhesion kinase- (FAK-) related cytosolic kinase, NOX4, SHP-2, Grb2, Janus kinase 2 (Jak2), proline-rich tyrosine kinase 2 (Pyk2), and integrin-associated protein (IAP) (Oshima et al., 2002; Maile et al., 2008; Shen et al., 2009; Xi et al., 2013). Among them, IAP is a transmembrane protein associated with several integrins, including the broadly expressed RGD receptor αvβ3, the platelet-fibrinogen receptor αIIbβ3, and the collagen receptor α2β1. In IGF-1 signaling, the SHPS-1-mediated association of IGF-1R with SHP-2 and Src kinase regulates the lifetime of phosphorylated IGF-1R and the duration of IGF-1 signaling. The association of IGF-1R with IAP enables the crosstalk between IGF-1R and integrin/FAK signaling, by which growth factor stimulation can be coupled with cell–ECM interactions (Maile et al., 2003).

NOX4 is not the only NOX family member that can bind to scaffold proteins involved in cytokine and integrin/ECM signaling. NOX2, for example, can be translocated to the cell front *via* the association of phosphorylated p47^phox^ with scaffold proteins such as tumor necrosis factor (TNF) receptor-associated factor 4 (TRAF4) and Wiskott–Aldrich Syndrome protein (WASP) family verprolin homologous protein 1 (WAVE1) (Wu et al., 2003; Li et al., 2005; Anilkumar et al., 2008; Kim et al., 2017; Fukai and Ushio-Fukai, 2020). Reciprocally, TNFα-induced Erk1/2 activation requires the association of phosphorylated p47^phox^ with TRAF4 (Li et al., 2005). Likewise, vascular EGF- (VEGF-) induced JNK activation needs the interaction of p47^phox^ with WAVE1 (Wu et al., 2003). The TRAF4-p47^phox^ association also plays an important role in the TRAF4-mediated thrombosis, suppressed by NOX2 inhibition (Arthur et al., 2011). These lines of evidence suggest not only spatial confinement of X-ROS to the vicinity of signaling targets (as the lifetime of ROS is short (Marklund, 1976)) but also crosstalk between X-ROS and cytokine/ECM signaling that leads to feedback amplification or suppression. The feedback signal could occur at multiple levels, including genetic regulations (e.g., TGFβ, insulin, and IGF-1 are known to increase NOX4 expression (Ning et al., 2002; Meng et al., 2008; Schroder et al., 2009; Kim et al., 2012; Watson et al., 2016; Liu W. J. et al., 2021; Yazaki et al., 2021)), PTMs (e.g., phosphorylation- and oxidation-mediated regulations), and ligand-receptor interactions. One example of ligand-receptor interactions is TGFβ signaling, where ROS produced by NOX4 promotes the activation of latent TGFβ, an inactive form of TGFβ secreted and bound to ECM (Watson et al., 2016). The activated ligands, in turn, can stimulate not only the ROS-producing cells but also nearby non-ROS-producing cells, leading to a multi-scale (i.e., autonomous and non-autonomous) niche response in the TGFβ-ROS signaling. Another example is integrin, the most abundant receptor for cell–ECM interactions. Growing evidence suggests that integrins are redox-sensitive, and NOX4-derived ROS can activate integrins through the cleavage of integrin α subunits (Wang et al., 1997; Ushio-Fukai, 2009; de Rezende et al., 2012; Eble and de Rezende, 2014).

### Module Box IV: Mechanics for the Regulation of Organ Size and Shape

To control cell shape and tissue topology, for decades, the dogma has been the interactions of diffusive extracellular cytokines and intracellular signaling molecules. It is suggested that the dynamic instability of interacting molecules can create spatiotemporal patterns to direct the assembly and remodeling of cytoskeleton and ECM, the mechanical output of which shapes cell and tissue boundaries and, in turn, determines cell fate. Conversely, little is known about whether the shape of cell and tissue boundary can spontaneously emerge through the mechanical instability of cytoskeleton and ECM and, in turn, direct the spatiotemporal patterns of signaling molecules and cytokines. Regardless of whether the chemical or the mechanical factors serve as the initial cues, cells need to continuously produce and respond to mechanical forces for the creation and maintenance of cell shape and tissue topology and often do so in synchrony with other cells (Cai D. et al., 2014). Unlike chemical signals, mechanical forces lack specificity and can be integrated, independent of the origins. Further, forces can be transmitted between and across cells through cytoskeletons, membranes, intercellular adhesions (Ragsdale et al., 1997; Vaezi et al., 2002), and ECM (Reinhart-King et al., 2008). Unlike the isotropic diffusion of cytokines, the transmission of forces within the boundary depends on the topology and structure of materials and hence can be fast, long-range, and highly anisotropic. Cells can likely take advantage of these properties to create long-range regulators and/or communicators. In fact, it has been shown that cells use membrane tension as a long-range inhibitor to regulate their polarization and morphology (Toriyama et al., 2010; Houk et al., 2012). We have also shown that epithelial cells create forces at collagen-based ECM and use them as a long-range coordinator to guide the self-assembly of tubular patterns (Guo et al., 2012).

Following the laws of thermodynamics, cell shape and tissue topology are determined by minimizing the surface free energy, which creates local forces at the boundaries, such as shear and/or normal stresses, that in turn evoke signaling activities to change cell fate. Shear stress, for example, is known to facilitate the respiratory barrier function and renal tubulogenesis, and failure in these processes leads to an abnormality such as polycystic kidney diseases (Sidhaye et al., 2008; Cattaneo et al., 2011). Similarly, normal stress that stretches the boundary between cells and ECM can lead to cell proliferation, whereas compression can lead to stem cell differentiation, as in the formation of teeth and cartilages (Terraciano et al., 2007; Mammoto et al., 2011; Aragona et al., 2013). In both cases, the forces are transduced into chemical signaling, such as the expression or nuclear translocation of transcriptional factors, Pax9, Sox-9, and/or YAP (Terraciano et al., 2007; Dupont et al., 2011; Mammoto et al., 2011). In this regard, cell mechanics and cytokine signaling appear to be both upstream and downstream of each other, with cell mechanics as a double-edged sword to facilitate organ development and potentiate cancer progression.

From the physics perspective, cell mechanics contains the passive components and the active elements, corresponding to the mechanical structures/properties of cells and the forces created therein, respectively. In general, forces created or acting at the cell include isotropic ones, such as osmotic pressure regulated by ion channels/pumps and water channels, and anisotropic ones, such as shear stress, cytoskeletal polymerization-mediated expansion, actomyosin-mediated retraction, and adhesions at the cell–cell and cell–ECM interfaces. For osmotic pressure, one example is the NOX2-mediated activation of ENaC, in which NOX2 produces ROS to activate the nearby ENaC (*via* cysteine oxidization) and induce sodium influx (Takemura et al., 2010; Goodson et al., 2012). A similar effect has been found in peroxynitrite (OONO^−^, created by NO + O_2_^−^) mediated inhibitory cysteine glutathionylation on the sodium-potassium pump (Na^+^-K^+^ ATPase), which causes intracellular sodium retention (Brown and Griendling, 2009; Figtree et al., 2009). The increment of intracellular sodium concentration, in turn, brings water into the cell through the water channels and aquaporin and induces calcium influx through the reverse mode of the sodium-calcium exchanger (NCX) (Tykocki et al., 2012; Ma and Liu, 2013; Yan et al., 2015; Chifflet and Hernandez, 2016) (Figures 3B,C). Depending on the cell type, calcium influx can activate NOX5, Duox1-2 (Brandes et al., 2014b; a), and/or PKC (Shirai and Saito, 2002), which can phosphorylate p47^phox^, p67^phox^, p40^phox^, Rac, and NOX2 (Cathcart, 2004; Brown and Griendling, 2009; Gorlach et al., 2015), leading to a positive feedback amplification on ROS-Ca^2+^ signaling. Calcium and the activated Rac1 can further promote actomyosin association and actin filament polymerization, respectively, thereby connecting the feedback with cell mechanics.

Rac1 is not the only ROS-activated effector in cytoskeletal dynamics. SrcFK, for example, can be activated by ROS through cysteine oxidization to phosphorylate the Rho GEF ARHGEF1 and the Rho-associated protein kinase (ROCK), thereby promoting RhoA activation for actin filament polymerization and myosin light chain (MLC) phosphorylation for actomyosin contraction (MacKay et al., 2017). *Via* cysteine oxidization, ROS also enables the association of Ras GTPase-activating-like protein or IQ motif-containing GTPase activating protein 1, IQGAP1, with NOX2 and cytokine receptors such as VEGF receptor (VEGFR) at the lamellipodial leading edge (Ikeda et al., 2005; Kaplan et al., 2011) (Figure 4A). IQGAP1-3 are scaffold proteins interacting with more than 100 molecules. These molecules include CD44, Rac1, Cdc42, formin mDia1, inverted formin-2 (INF-2), WASP, microtubule plus-end binding protein CAP-GLY domain-containing linker protein 1 (CLIP1), and cytoplasmic linker associated protein 1 (CLASP1), adenomatous polyposis coli (APC), β-catenin, Mek1/2 kinase, Erk1/2, Src kinase, integrin-linked kinase (ILK), 5ʹ AMP-activated protein kinase (AMPK), PTP, and ezrin (Brandt et al., 2007; Le Clainche et al., 2007; Watanabe et al., 2009; Malarkannan et al., 2012; White et al., 2012; Widmaier et al., 2012; Liu et al., 2014; Smith et al., 2015; Bartolini et al., 2016; Sayedyahossein et al., 2016; Hedman et al., 2021). IQGAPs also bind to CLASP2, YAP, and the regulators of YAP in the Hippo pathway, MST2, and LATS1 (Watanabe et al., 2009; Sayedyahossein et al., 2016; Quinn et al., 2021). Through these binding capacities, the cytokine-NOX2 signaling can confine microtubule plus end, ROS signals, kinase activities, and actin filament polymerization at the cell leading edge, by which it not only directs the microtubule transport-delivered surface receptors and signaling molecules to the moving front but also interferes with YAP-dependent mechanotransduction.

IQGAP1 is involved in microtubule dynamics. To date, the most well-studied system for the coupling of X-ROS and microtubule dynamics is the cardiomyocytes. These cells are huge (with cell volume ≥40,000 μm^3^), in a rod shape packed with dense cytoskeletal networks that can be divided into two groups—the contractile actomyosin arrays organized into myofibrils and the viscoelastic microtubule bundles aligned in the longitudinal direction of the cells. Given the long persistence length of microtubules (∼0.5–1.5 mm^2^ (Gittes et al., 1993; van Mameren et al., 2009)), it is plausible that microtubules serve as the mechanical sensor to detect the conformational change of the cell as a whole. In fact, it was shown that physiologic stretch elicits a rapid activation of NOX2 in these cells, likely through the release of microtubule-bound Rac1-GTP (Best et al., 1996) by mechanical deformation to activate nearby NOX2. NOX2-derived ROS then sensitize nearby sarcoplasmic reticulum (SR) calcium channels, ryanodine receptors (RyRs), by cysteine oxidation to release calcium ions in response to the mechanical stretch as a rapid and localized mechano-chemo transduction process (Prosser et al., 2011). Conversely, in muscle contraction, microtubules buckle to bear the mechanical load created by the actomyosin contraction. The buckling not only elicits X-ROS signaling but also provides resistance against the contraction (Robison et al., 2016). The amount of elicited X-ROS signals depends on the PTMs of microtubules. It was shown that detyrosinated microtubules, a stable microtubule subpopulation, are responsible for muscle stiffness and X-ROS generation during contraction (Robison et al., 2016). As a result, suppressing microtubule detyrosination provides a therapeutic strategy to treat patients with hypertrophic or dilated cardiomyopathies, both of which carry a higher amount of detyrosinated microtubules than normal (Chen et al., 2018).

With the cytoskeletal dynamics and NOX activity mutually influencing each other, it is plausible that NOX is subject to the mechanical modulation in cell morphogenesis and involved in cell mechanotransduction. Indeed, recent studies have shown that cyclic stretch increases mitochondria-released ROS, FAK phosphorylation at Tyr397, and PKC activity (Ali et al., 2006). PKC and the released ROS, in turn, activate (through phosphorylation and/or cysteine oxidization) p47^phox^, p67^phox^, p40^phox^, Rac, NOX2, SrcFK, and NOX4 (Shirai and Saito, 2002; Cathcart, 2004; Brown and Griendling, 2009; Xi et al., 2013; Gorlach et al., 2015) to enhance ROS production, whereas FAK recruits Src kinase to the integrin cytoplasmic tails and forms a complex therein to induce multiple responses including PI3K-AKT activation, actin filament polymerization, and focal adhesion complex formation (Bolos et al., 2010; Zhao and Guan, 2011). The cysteine-oxidized SrcFK then activates ARHGEF1 and ROCK to enhance MLC phosphorylation, stress fiber assembly, and force generation at the cell–ECM interface (Tominaga et al., 2000; MacKay et al., 2017), by which the mechanical stretch between cells and the ECM could be reinforced (Figure 2C). In addition, mechanical stretch can induce persistent calcium influx *via* microtubule-dependent activation of NOX2 to generate ROS, which acts on redox-sensitive transient receptor potential (TRP) channels (Song et al., 2011; Taylor-Clark, 2016; Pires and Earley, 2017; Pratt et al., 2020; Singh et al., 2021) such as TRPA1, TRPM2, TRPV4, and TRPC6 to evoke or prolong calcium signaling, thereby enhancing PKC activity (Shirai and Saito, 2002) and actomyosin assembly and contractility (through, e.g., activating the CaM (calmodulin)/MLCK-signaling pathway (Zergane et al., 2021)). The integration of these effects can lead to a self-perpetuated amplification of the cellular mechanical responses, which might serve as a switch for the selection of stem cell fate. One example is the cyclic stretch-induced cardiomyogenesis of mouse embryonic stem cells in the presence of Wnt/β-catenin signaling (Heo and Lee, 2011). At the genetic level, mechanical stretch can modulate NOX and HIF-1α expressions (Grote et al., 2003; Schmelter et al., 2006; Sauer et al., 2008; Zhang et al., 2015). However, the effect is exposure time- and pattern-dependent (Goettsch et al., 2009) and can lead to positive or negative feedback regulations, a detailed review of which can be found elsewhere (Brandes et al., 2014a).

### Module Box V: Merlin, YAP, and Angiomotin as Transducers for Cell Mechanics and Tissue Topology

YAP/TAZ boost organ growth and are suppressed by the Hippo pathway (Wang et al., 2009; Zhao et al., 2010a; Li et al., 2010; Lian et al., 2010; Dupont et al., 2011; Yu et al., 2015; Panciera et al., 2017; Totaro et al., 2018b). YAP is referred to as WW domain-containing transcription coactivator Yes-associated protein (Sudol, 1994), TAZ is referred to as transcriptional coactivator with PDZ-binding motif, also known as WW domain-containing transcription regulator 1 (WWTR1) (Sarmasti Emami et al., 2020), and the Hippo pathway, also known as the Salvador–Warts–Hippo (SWH) pathway, is a pathway that controls organ size by restraining cell proliferation and promoting apoptosis (Piccolo et al., 2014). Herein, PDZ stands for post-synaptic density 95, Discs large, and Zonula occludens-1, whereas the WW domain, named after the presence of two tryptophan (W) residues and also known as the rsp5-domain or WWP repeating motif, is a modular protein domain preferentially binding to proline-rich, for example, PPXY and LPXY, or phosphor-serine/threonine-containing (e.g., p-SP/p-TP) motifs (Chen and Sudol, 1995; Sudol et al., 1995; Macias et al., 1996; Lu et al., 1999). YAP/TAZ has a critical role in stem cell self-renewal and tissue-specific progenitor cell self-expansion (Dong et al., 2007; Zhao et al., 2011b; Anton and Wandosell, 2021), where YAP/TAZ is accumulated and active in the cell nucleus (Camargo et al., 2007; Cao et al., 2008; Schlegelmilch et al., 2011; Silvis et al., 2011; Lavado et al., 2013). Moreover, as hyperactive YAP/TAZ leads to uncontrolled cell growth, a growing interest has been raised in the roles of YAP/TAZ in cancer progression (Saucedo and Edgar, 2007; Xu et al., 2009; Pan, 2010; Johnson and Halder, 2014; Mo et al., 2014; Lee Y. A. et al., 2018). In fact, YAP/TAZ contributes not only to tumor growth but also to drug resistance (Lai et al., 2011; Zhao and Yang, 2015). Likewise, self-sustained YAP activity has been found in CAFs, through mutually enhanced cell contractility and “inside-out” ECM stiffening, to remodel the niche mechano-environment (i.e., the tumor microenvironment (TME)), thereby promoting tumor progression (Calvo et al., 2013; Piccolo et al., 2014). To date, the regulation of YAP/TAZ has been intensively studied. Herein, we focus on X-ROS-dependent cytokine/ECM signaling in the regulation of the Hippo pathway. In order to proceed, a short introduction of the Hippo pathway is given below. More details of this pathway can be found elsewhere (Pan, 2010; Yu and Guan, 2013; Piccolo et al., 2014; Zanconato et al., 2016a; Zanconato et al., 2016b; Meng et al., 2016; Panciera et al., 2017; Zheng and Pan, 2019; Sarmasti Emami et al., 2020; Zhao et al., 2020; Wang et al., 2021).

The Hippo pathway is processed by several serine/threonine kinases and cofactors in a multiplexed, sequential manner. These molecules include the mammalian Ste20-like protein kinase 1/2 (MST1/2), the Salvador family WW domain-containing protein 1 (SAV1), the large tumor suppressor kinase 1/2 (LATS1/2), and the Mps one binder (MOB) kinase activator-like 1 (MOB1) (Piccolo et al., 2014; Sarmasti Emami et al., 2020) (Figure 4B). The signaling starts from the association of MST1/2 with SAV1 into a hetero-tetramer complex (2 MST and 2 SAV1), by which MST1/2 perform auto-activation (at T180). Activated MST1/2-SAV1 then phosphorylate MOB1 and LATS1/2 (in a complex form) to induce LATS1/2 auto-phosphorylation and auto-activation (LATS1 at T1079 and LATS2 at T1041 (Ma et al., 2019; Sarmasti Emami et al., 2020)). Other kinases that act in parallel to MST1/2 and activate LATS1/2 include mitogen-activated protein kinase kinase kinase kinases (MAP4Ks) (Meng et al., 2015) and serine/threonine kinase 25 (STK25) (Lim et al., 2019). Once activated, LATS1/2 phosphorylate YAP at S61, S109, S127, S381, and S397 (Zhao et al., 2010b; Mo et al., 2014; Piccolo et al., 2014; Ni et al., 2015; Elisi et al., 2018; Mana-Capelli and Mccollum, 2018; Sarmasti Emami et al., 2020), which is counteracted by the protein phosphatase magnesium-dependent 1A (PPM1A or PP2Cα) (Zhou et al., 2021), PP1A (Li et al., 2016), and PP2A (Schlegelmilch et al., 2011), or the pre-phosphorylation of YAP at S128 by Nemo-like kinase (NLK) (Hong et al., 2017; Moon et al., 2017). YAP with phosphorylation at S127 is a target of 14-3-3 proteins, whereas phosphorylation at YAP S381 or S397 creates a phosphor-degron motif for the subsequent phosphorylation by casein kinase 1 (CK1) and binding of Skp1-Cullin-1-F-box protein (SCF) type of E3 ubiquitin ligase, SCF^β-TrCP^, which catalyzes the ubiquitination and degradation of YAP (Hao et al., 2008; Zhao et al., 2010b; Liu et al., 2010; Iwasa et al., 2013; He et al., 2016). The association of YAP with 14-3-3 proteins sequesters YAP in the cytoplasm or at the adherens junctions (AJs) (*via* the association of AJ α-catenin with 14-3-3 proteins and YAP (through its WW-domain)) (Schlegelmilch et al., 2011; Yu and Guan, 2013). In epithelial organs, 14-3-3 protein-potentiated association of YAP with α-catenin depends on the cell density and the maturation of AJs. When the cells are at low-density states or with immature AJs, α-catenin fails to sequester YAP at AJs, and the cytoplasmic 14-3-3 protein-YAP complex is subject to the PPM1A/PP2A-mediated dephosphorylation at YAP S127 (Schlegelmilch et al., 2011; Zhou et al., 2021).

14-3-3 proteins are not the only molecules to sequester YAP. Switch/sucrose non-fermentable (SWI/SWF), an ATP-dependent chromatin remodeling complex, can bind to YAP in the nucleus through AT-rich interactive domain-containing protein 1A (ARID1A), thereby inactivating the transcriptional activity of YAP (Chang et al., 2018). Dishevelled (DVL), a scaffold molecule in the Wnt pathway (Barry et al., 2013), can sequester pS127-YAP in the cytoplasm (Lee Y. et al., 2018). Angiomotin (AMOT), a PDZ domain-binding protein, can bind to and sequester YAP in the cytoplasm and/or at the tight junctions (TJs), but the association acts through the YAP WW domain without YAP S127 phosphorylation (Zhao et al., 2011a; Yi et al., 2013; Moleirinho et al., 2017; Wang et al., 2021) and depends on actin dynamics because actin filaments and YAP compete for the same binding site at AMOT (Yi et al., 2011; Li et al., 2015). Likewise, protein tyrosine phosphatase non-receptor type 14 (PTPN14) can bind to YAP through the WW domains of YAP and sequester YAP at AJs or in the cytoplasm without YAP S127 phosphorylation (Poernbacher et al., 2012; Wilson et al., 2014). Other molecules that can sequester YAP without YAP S127 phosphorylation include axin (Azzolin et al., 2014) and IQGAP1 (Quinn et al., 2021), both of which are the scaffold molecules for β-catenin. Axin, a scaffold that assembles APC, β-catenin, and GSK3β into the destruction complex of β-catenin in the Wnt signaling pathway, can bind to and sequester YAP in the cytoplasm (Azzolin et al., 2014) or the multi-vesicular body (MVB) (Gargini et al., 2016; Rivas et al., 2018; Anton and Wandosell, 2021). In comparison, the association of IQGAP1 and YAP occurs through the DNA-binding domain for the transcriptional enhancer factor TEF-1, TEC1, and AbaA (TEA domain- (TEAD-)) binding domain of YAP and does not explicitly sequester YAP into the nucleus or the cytoplasm. Instead, its major effect is to block YAP-TEAD nuclear interaction (Sayedyahossein et al., 2016). A similar mechanism is AMPK-mediated phosphorylation on YAP S94, which disrupts the YAP-TEAD association (Mo et al., 2015) in metabolic and nutrient-sensing regulations (Santinon et al., 2016). Intriguingly, IQGAP1 can bind to and suppress the activity of MST2 and LATS1 (Quinn et al., 2021), and as a result, it suppresses both the Hippo pathway and YAP signaling (Figures 4B,C).

YAP sequestered by 14-3-3 proteins or β-catenin destruction complex in the cytoplasm is subject to degradation *via* the ubiquitin-proteasome pathway (Zhao et al., 2020). By contrast, YAP molecules sequestered in the MVB, with IQGAP1, or at the AJs/TJs are prevented from degradation or nuclear translocation. Only free YAP (with or without S127 phosphorylation) can translocate into the nucleus for the transcription of target genes (Zhao et al., 2020) such as PD-L1 (Janse van Rensburg et al., 2018), connective tissue growth factor (CTGF), fibroblast growth factor 1 (FGF1), receptor tyrosine kinase AXL, BMP4, and pro-apoptotic or pro-survival genes (Kim M.-K. et al., 2018; Sarmasti Emami et al., 2020; Quinn et al., 2021). These genes are involved in not only organ development but also tumorigenesis, including enhanced cell migration and immune evasion. The nuclear accumulation of YAP, however, is counteracted by the neurofibromatosis type 2 (NF2, a 4.1 protein, ezrin, radixin, and moesin (FERM) domain-containing molecule, also known as moesin/ezrin/radixin-like protein (Merlin) or schwannomin (Bretscher et al., 2002; Baser et al., 2003; McClatchey, 2003)) which exports YAP out of the nucleus *via* its nuclear localization signal (NLS) sequence and nuclear export signal (NES) sequences (Gladden et al., 2010; Furukawa et al., 2017). Such Merlin-assisted nuclear export of YAP acts independently of the Hippo pathway or other related molecules such as AMOT. Instead, it requires cells at high densities or with high intercellular tension (Furukawa et al., 2017). Another molecule for YAP nuclear export is DVL, which acts only when YAP S127 is phosphorylated (Barry et al., 2013; Lee Y. et al., 2018).

These lines of evidence indicate that the regulation of the Hippo pathway and YAP signaling occurs through PTMs (e.g., phosphorylation and ubiquitination) and compartmentalization. The question is how these processes are linked to the organ size and shape, or more explicitly, to the ROS-dependent cytokine/ECM signaling and the cell mechanics in organ development, repair, and malignancy. Mechanistically, MST1 and MST2 share functional redundancy. They contain an N-terminal kinase domain and a C-terminal coiled-coil SAV/Ras-association domain family (RASSF)/HPO (SARAH) domain with a flexible linker in between (Jin et al., 2012; Ni et al., 2013). SARAH domains are self-associable. Through SARAH-domain self-association, MST1/2 form homodimers and undergo trans-autophosphorylation at T180 (in the kinase domain) and at T325, T336, and T378 (in the linker region) (Bae et al., 2017). The trans-phosphorylation of T180 leads to MST1/2 auto-activation. The trans-phosphorylation of the linker, however, inhibits MST1/2 by recruiting a multi-subunit PP2A complex, striatin- (STRN-) interacting phosphatase and kinase (STRIPAK), through an adaptor, sarcolemmal membrane-associated protein (SLMAP), to dephosphorylate T180 and counteract MST1/2 auto-activation (Bae et al., 2017). Initially defined as a non-canonical PP2A regulatory subunit (B subunit) (Moreno et al., 2000), STRN has a caveolin-binding domain, a coiled-coil domain, a Ca^2+^-calmodulin- (CaM-) binding domain, and a tryptophan–aspartate- (WD-) repeat domain, by which it can recruit and bind to multiple partners (Hwang and Pallas, 2014). The resulting complex, STRIPAK, contains a PP2A catalytic subunit (PP2AC), scaffolding subunit (PP2AA), and the STRN regulatory subunit that recruits STRN-interacting protein (STRIP1/2), SLMAP, and members of the STE20 family of kinases (e.g., MST1/2) (Couzens et al., 2013).

The ability to auto-activate and recruit inhibitors to deactivate itself at the same time, as in the case of MST1/2 and STRIPAK, is not rare in biology. POPX2, for example, forms a trimeric complex with the Rac1/Cdc42 guanine nucleotide exchange factor ARHGEF7 (also known as the p21-activated protein kinase-exchange factor β (βPIX)) and PAK, wherein Rac1-activated PAK is subject to immediate dephosphorylation by POPX2 (Kim P. R. et al., 2020). Another example is the complex formation of the scaffold molecule, muscle-selective A-kinase anchoring proteins (mAKAP), with cAMP-specific phosphodiesterase (PDE)-4D3 (PDE4D3) and PKA, wherein the PKA activity is subject to the downregulation of cAMP level by PDE4D3 (Sette and Conti, 1996; Lim et al., 1999; Rababa'h et al., 2013). From the thermodynamics point of view, having the auto-activation and auto-inhibition occur at the same time places the complex in a highly unstable state. However, from the evolutionary point of view, this scenario provides an ability to create instant Hippo “on/off” signals in response to tissue injury or remodeling. The inhibitory effect of STRIPAK on MST1/2 is antagonized by the association of SAV1, which contains an N-terminal flexible region, a tandem repeat of two WW domains, and a C-terminal SARAH domain (Bae et al., 2017). The N-terminal region of SAV1 has a FERM domain-binding motif to bind to FERM-domain proteins such as Merlin and Expanded (Ex) (Bretscher et al., 2002; Baser et al., 2003; McClatchey, 2003) and a protein interaction domain (PID) to bind to and suppress STRIPAK (Bae et al., 2017). Similar to the SARAH domains, the WW domains of SAV1 are self-associable (Ohnishi et al., 2007) but act through a domain-swapping mechanism between two SAV1s (Lin et al., 2020). *Via* the intermolecular association of SARAH and WW domains, two SAV1 and two MST1/2s can form a hetero-tetramer, thereby bringing the N-terminal of SAV1 to the proximity of STRIPAK to antagonize the phosphatase. However, the binding affinity between the SAV1 N-terminal and STRIPAK is low (with *K*_*m*_ ∼100 μM (Bae et al., 2017)). Additional modulators are thus needed to facilitate the suppression.

The AJ- and TJ-associated factors, including WW domain and C2 domain-containing proteins (WWC), such as kidney and brain expressed protein (KIBRA /WWC1 (Hoffken et al., 2021)); PDZ-domain proteins, such as AMOT; and FERM domain proteins, such as Merlin, Ex, and PTPN14 appear to be the key modulators. Predominantly expressed in the kidney and the brain, the first key molecule, KIBRA, contains two WW domains (Kremerskothen et al., 2003), a potential coiled-coil domain, a C2 domain responsible for Ca^2+^-sensitive interaction with phospholipids, a class III PDZ-binding motif ADDV, and an atypical protein kinase C (aPKC) binding region (Baumgartner et al., 2010; Genevet et al., 2010; Yu et al., 2010; Boggiano and Fehon, 2012; Zhang et al., 2014; Su et al., 2017). More than 20 binding partners of KIBRA have been identified. These molecules include Merlin, Ex, FRM6 (FERM domain-containing protein 6), AMOT, PALS1- (protein associated with Caenorhabditis elegans Lin-7 protein 1-) associated tight junction protein (PATJ), PTPN14, PP1, SAV1, LATS1/2, the mitotic serine/threonine kinase Aurora-A, PKCζ, and the apical polarity complexes PAR3/PAR6β (partition-defective 3/partition-defective 6β) (Xiao et al., 2011a; Xiao et al., 2011b; Zhang et al., 2014; Zhou et al., 2017). At mature TJs, KIBRA forms a complex with Merlin, Ex, and AMOT to interact with MST1/2-SAV1 and LATS1/2-MOB1, thereby promoting LATS auto-activation and YAP phosphorylation (Zhang et al., 2014) (Figure 4D). By having such a complex formation, Merlin can likely be placed at the SAV1-STRIPAK binding interface, thereby stabilizing SAV1-STRIPAK interaction and suppressing STRIPAK activity (Bae et al., 2017). However, the association of Merlin with KIBRA is inhibited by Aurora-mediated phosphorylation at KIBRA S539, which is counteracted by PP1 and PTPN14 (Xiao et al., 2011b; Poernbacher et al., 2012; Wang W. et al., 2014), suggesting a link between cell mitosis and the Hippo pathway. In the presence of apicobasal polarity, KIBRA-Merlin-FRMD6 complex formation competes with Par3-aPKC- KIBRA complex formation (Suzuki and Ohno, 2006; Yoshihama et al., 2009; Zhou et al., 2017), and KIBRA directly suppresses aPKC and aPKC-mediated apical exocytosis (Yoshihama et al., 2011), by which cells can limit the expansion of apical surface, an important feature in stem cell homeostasis and absent in tumorigenesis.

The second key molecule, AMOT, is an AJ/TJ-associable PDZ-domain protein that plays an important role in regulating the partitioning of Merlin and YAP, as shown in the differentiation of human pluripotent stem cells (Zaltsman et al., 2019). AMOT possesses an N-terminal domain, which contains a WW domain- and actin-binding motif (157–191) that YAP and actin filaments compete binding to, followed by a coiled-coil domain that can bind to Merlin (Yi et al., 2011; Li et al., 2015), and a C-terminal PDZ domain that can bind to TJs (Hirate and Sasaki, 2014; Mana-Capelli et al., 2014). The competition of YAP and actin filaments with AMOT appears to depend on the “structural code” of the cells. In blastocysts, for example, the embryonic cells are segregated into an outer layer with cells forming apicobasal polarity and an inner layer without polarity formation. AMOT localizes to the AJs of non-polarized cells at the inner layer, with S176 phosphorylated by LATS, which inhibits actin binding, stabilizes the AMOT-LATS interaction (Dai et al., 2013; Hirate et al., 2013; Hirate and Sasaki, 2014), promotes AMOT-YAP association, and enables YAP phosphorylation (Mana-Capelli et al., 2014). By contrast, AMOT is unphosphorylated and sequestered to the apical actin at the outer layer, thereby releasing YAP for nuclear signaling (Hirate et al., 2013) (Figure 4D).

The third key molecule, Merlin, contains an N-terminal FERM domain (sequence 19–313), followed by one α-helical domain (314–507) and one C-terminal domain (508–595) (Muranen et al., 2007; Li et al., 2015). The α helical domain possesses a coiled-coil motif that can bind to AMOT (Yi et al., 2011; Li et al., 2015), whereas the N-terminal and the C-terminal can self-associate (McClatchey, 2003) with *K*_*m*_ ∼3 μM, a much weaker affinity than that of ERM proteins (*K*_*m*_ ∼0.016 μM) (Li et al., 2015). Merlin has many binding partners (Hennigan et al., 2019) through which it can associate with or dissociate from AJs. The selection of binding partners is primarily modulated by Merlin PTMs such as phosphorylation and ubiquitination (Laulajainen et al., 2011). These PTMs displace the self-associated C-terminal and N-terminal of Merlin away from each other, thereby exposing the binding sites to, for example, MST1/2-SAV1, LATS1/2, YAP, AKT, paxillin, FAK, and integrin β1 (Obremski et al., 1998; Fernandez-Valle et al., 2002; James et al., 2004; Tang et al., 2007; Yamauchi et al., 2008; Flaiz et al., 2009; Yi et al., 2011; Yin et al., 2013; Li et al., 2015; Bae et al., 2017). Alternatively, Merlin can bind to PIP_2_ through its FERM domain, by which Merlin adopts an expanded conformation to expose the binding sites (Ali Khajeh et al., 2014). Thus, Merlin “with” and “without” PTMs (and/or PIP_2_ binding) are often referred to as the “open” and “close” states, respectively. Historically, in the “close” state, Merlin has been known as the tumor suppressor (Li et al., 2015). For the “open” state, major phosphorylation sites include S10 and S518 by PKA (Laulajainen et al., 2008); S10, T230, and S315 by AKT (Tang et al., 2007; Laulajainen et al., 2011); and S518 by PAK (Shaw et al., 2001). Phosphorylating Merlin at S518 prevents Merlin from participating in the Hippo pathway and sequesters Merlin on the cell membrane through the association with the C-terminal of cell surface receptors such as CD44 (Morrison et al., 2001; Sherman and Gutmann, 2001) and/or bind to tubulin to enhance microtubule polymerization (Muranen et al., 2007). Merlin S518 phosphorylation is counteracted by myosin phosphatase target subunit 1- (MYPT1-) regulated PP1c, the phosphatase for MLC (Jin et al., 2006). MYPT1 is inactivated by ILK or ROCK-mediated phosphorylation at T696 (by ILK/RCOK) and S854/T855 (by ROCK) (Serrano et al., 2013; Hartmann et al., 2015), which occurs during cell migration or spreading on stiff substrates. Alternatively, MYPT1 can be sequestered by phosphorylated MLC when cells are spreading on stiff substrates (Joo and Yamada, 2014). These lines of evidence provide an explanation for how stiff microenvironments might disable Merlin-mediated tumor suppression, enhance YAP nuclear translocation, and promote tumor invasion (Paszek et al., 2005; Guo et al., 2012).

In contrast to phosphorylation at S518, Merlin phosphorylated at S10, T230, and/or S315 is subject to ubiquitination and proteasome-mediated degradation (Laulajainen et al., 2011), which, however, requires S518 to be dephosphorylated (Li et al., 2015; Wei et al., 2020), suggesting that Merlin phosphorylated at S518 and Merlin phosphorylated at S10, T230, and/or S315 are two functionally exclusive states (Figure 4D). Merlin ubiquitination is mediated by the E3 ubiquitin ligase, neural precursor cell expressed developmentally downregulated protein 4 (NEDD4), which conjugates one or two ubiquitin molecules at Merlin K396 and K159 by the aid of AMOT (Wei et al., 2020). In this process, AMOT serves as a scaffold protein to bind to Merlin through their mutual coiled-coil domains (Yi et al., 2011; Li et al., 2015) and bind to NEDD4 through the association of its two PPXY motifs with the WW domains of NEDD4 (Skouloudaki and Walz, 2012). Although Merlin ubiquitination promotes the degradation of the Merlin-AMOT complex, it is required for MST-mediated LATS phosphorylation (Wei et al., 2020). The ubiquitinated Merlin-AMOT complex can bind to the N-terminal FERM-binding domain (FBD) of LATS through the Merlin FERM domain (*K*_*m*_ ∼1.4 μM (Yin et al., 2013; Li et al., 2015)). Other associations include those between SAV1 and MOB-1 (Yin et al., 2013), between the Merlin FERM domain and the N-terminal FERM domain-binding motif of SAV1 (Bretscher et al., 2002; Baser et al., 2003; McClatchey, 2003; Bae et al., 2017), between the extreme N-terminal end of Merlin and α-catenin (Cole et al., 2008), and between AMOT and YAP (at the N-terminal actin-binding motif of AMOT (157–191)) (Mana-Capelli et al., 2014). Through these molecular associations, the ubiquitinated Merlin-AMOT complex likely promotes the clustering of YAP, LATS-MOB1, and MST-SAV1 at AJs (by Merlin-α-catenin-AJ association) or TJs (by AMOT PDZ domain-TJ association), wherein MST1/2 potentiate LATS1/2 auto-activation (Gladden et al., 2010; Hansen et al., 2015) (Figures 4B,D). In addition, independent of MST, AMOT can act along with MOB1 to promote LATS autophosphorylation and auto-activation (Mana-Capelli and Mccollum, 2018). Activated LATS phosphorylates not only YAP but also AMOT (at S175/S176), the phosphorylation of which suppresses actin binding to AMOT and stabilizes the binding between LATS-MOB1 and Merlin-AMOT (Dai et al., 2013; Hirate and Sasaki, 2014; Mana-Capelli et al., 2014; Moleirinho et al., 2017; Mana-Capelli and Mccollum, 2018) (Figure 4D). As a result, the Merlin-AMOT complex can likely promote LATS activation and YAP phosphorylation in a self-sustained manner.

The fourth key molecule, an AJ-associable FERM domain protein for YAP modulation, is PTPN14 (Wang et al., 2012; Huang et al., 2013; Liu et al., 2013; Wilson et al., 2014). PTPN14 contains an N-terminal FERM domain, followed by two PPXY motifs which are essential for the interactions with YAP and KIBRA through their WW domains (Poernbacher et al., 2012), and a C-terminal PTP catalytic domain which is essential to counteract Src kinase- or receptor tyrosine kinase- (RTK-) mediated phosphorylation at β-catenin Y654 and VE-cadherin, thereby stabilizing AJs (Van Veelen et al., 2011; Fu et al., 2020). Along with the PPXY motifs, the PTP domain of PTPN14 is required for the interactions with KIBRA (Wilson et al., 2014). Further, PTPN14 can bind to LATS1/2 and acts independently or cooperatively with KIBRA to enhance LATS1/2 auto-activation and YAP S127 phosphorylation, even in the absence of MST1/2 (Wilson et al., 2014). Thus, the KIBRA-AMOT-Merlin complex and KIBRA-PTPN14 complex can act in parallel to modulate YAP phosphorylation and sequestration at AJs (Figure 4D).

### Module BOX VI: The HIF/YAP/Notch Triad and PD-L1

YAP/TAZ forms complexes with HIF-1α and functions as the transcription activator of HIF-1α to enhance expressions of molecules involved in organ development, tissue homeostasis, and tumorigenesis (Xiang et al., 2015; Zhao et al., 2020). Under hypoxia, YAP binds to nuclear HIF-1α and sustains its stability, thereby promoting the expression of pyruvate kinase isozymes M2 (PKM2), a key enzyme of glycolysis, in, for example, hepatocellular carcinoma cells (HCCs) (Zhang X. et al., 2018), whereas in the cytoplasm, YAP enhances HIF-1α stability by inhibiting VHL-dependent degradation of hydroxylated HIF-1α (Ma et al., 2015; Zhao et al., 2020) (Figure 1A). Likewise, HIF-2α, another hypoxia responding subunit, has been found to increase YAP1 expression and activity, yet it does so without the involvement of Src kinase, PI3K, ERK, or MAPK signaling pathways (Ma et al., 2017). No direct association between YAP and HIF-2α was observed either (Ma et al., 2017). In addition to glycolysis, the YAP-HIF-1α complex promotes the transcription of genes involved in angiogenesis and cell growth (Zhao et al., 2020). Genes containing HREs that H1F-1α can bind to also include *WWTR1* (i.e., TAZ) and *SIAH1* (Zhao et al., 2020), and the TAZ-HIF-1α complex has been shown to promote the transcription of SIAH1 (Xiang et al., 2014; Xiang et al., 2015; Zhao et al., 2020). Similar to SIAH2 (Ma et al., 2015), SIAH1 induces LATS2 degradation and, in turn, TAZ nuclear localization (Xiang et al., 2014; Xiang et al., 2015; Zhao et al., 2020). Thus, positive feedback exists along the YAP/TAZ-HIF/SIAH axis (Figure 5A). In the development of growth plate, for example, HIF-1α was found to promote YAP activation and, in turn, upregulate the expression of sex-determining region-box 9tbox9 protein (SOX-9), a marker of stemness, for the maintenance of chondrogenic phenotype (Li H. et al., 2018).

Notch signaling is a highly conserved cell–cell communication mechanism by which cells regulate organ development, homeostasis, and repair through lateral inhibition (or “trans-inhibition”) between neighboring cells (Kopan and Ilagan, 2009; Guruharsha et al., 2012; Kovall et al., 2017; Siebel and Lendahl, 2017). Notch is a cell surface receptor. Upon ligand binding, Notch is cleaved to release Notch intracellular domain (NICD), which translocates into the nucleus to bind to CSL (the transcriptional repressor CBF1/suppressor of hairless/Lag-1) or the human homolog RBPJ (recombination signal-binding protein for immunoglobulin κJ region, also known as CBF1) to facilitate the transcription of Notch target genes (Kopan and Ilagan, 2009). In tumorigenesis, Notch signaling promotes the CSC formation by reducing their proliferation yet increasing their resistance to therapies, thereby potentiating cancer cell dormancy and relapse (Janghorban et al., 2018). Moreover, Notch has been proposed as a mechanical sensor based on the observation that Notch can be activated by mechanical stretch and shear stress (Gordon et al., 2015; Chowdhury et al., 2016; Mack et al., 2017; Loerakker et al., 2018) and that Notch participates in mechanics-dependent periodic feather branch pattern formation (Cheng et al., 2018). For proper organ development, the specification of cell fate must be spatiotemporally coordinated with tissue morphogenesis. Therefore, it is plausible that the signaling of the “messengers” for tissue structure and mechanics (i.e., YAP/TAZ) is linked to Notch signaling pathways, by which cells can sense the mechanical changes in the niche through (e.g., cell–ECM adhesions and cell–cell contacts) and make a correspondent decision on the cell fate.

Several examples support the idea that YAP/TAZ and Notch signaling pathways are coupled (Totaro et al., 2018a). This coupling can be positive or negative, with YAP/TAZ acting upstream of, downstream of, or in parallel with Notch signaling. Such versatility is achieved by having YAP/TAZ and Notch synergistically co-regulate shared target genes, having YAP/TAZ act upstream to regulate the expression of Notch ligands or receptors (thereby downregulating or upregulating Notch activity, respectively), or having Notch act upstream to upregulate or downregulate YAP activity (Totaro et al., 2018a). An example of the synergistic coupling is the control of smooth muscle differentiation from neural crest cells (Manderfield et al., 2015), where YAP/TAZ forms a complex with NICD to promote the transcription of Notch target genes for smooth muscle fate (Manderfield et al., 2012). Another example is the binary cell fate decision in the embryonic transition from morula to blastocyst. In this case, the binary decision occurs between cells becoming inner cell mass or outer-layer trophectoderm (TE) (Nishioka et al., 2009; Hirate et al., 2013; Leung and Zernicka-Goetz, 2013; Engel-Pizcueta and Pujades, 2021). In this case, Notch and YAP/TAZ act in parallel and non-redundantly to drive the specification of the TE fate gene, *Cdx2*, by having Notch elicit the onset of Cdx2 expression and YAP maintain the expression of Cdx2, respectively (Watanabe et al., 2017). For YAP/TAZ acting upstream to upregulate Notch, one example is the binary cell fate decision made between cholangiocytes and hepatocytes in liver development (Kodama et al., 2004; Zong et al., 2009). In this case, YAP drives *Notch2* expression and cholangiocyte specification and proliferation (Wu et al., 2017). Another is between the tip and stalk cells in angiogenesis, where YAP/TAZ suppresses the β-catenin-NICD-mediated expression of Notch ligand and endothelial Delta-like 4 (Dll4) protein in the tip cells (Yasuda et al., 2019). For YAP acting upstream to downregulate Notch, the example is the homeostasis of the epidermis, where Notch signaling is required for the transition of keratinocytes from the basal to the suprabasal layers (Siebel and Lendahl, 2017; Totaro et al., 2018a). In this case, the segregation of cell fate at different layers is achieved by spatially confining the Notch activity throughout the entire epidermis. Cells in the basal layer mainly express the Notch ligands Delta-like 1 (Dll1) and Jagged-2 (Jag2), whereas cells in the suprabasal layers mainly express the Notch receptors. Mechanical stretch and/or ECM stiffness in the basal layer activates YAP/TAZ signaling, which suppresses Notch activity by upregulating the expression of Notch ligands to counteract Notch activity through the “cis-inhibition,” that is, having the Notch ligand and receptor co-expressed on the same cell surface to suppress the Notch activity (Totaro et al., 2018a). Conversely, for Notch acting upstream of YAPTAZ, an example is the symmetric stem cell division in the embryonic brain development, where Notch upregulates YAP expression by the binding of NICD-RBPJ complex to the YAP promoter, thereby promoting neural stem cell symmetric proliferation (Li et al., 2012).

Recent studies on glioblastoma stem cells revealed the differential roles of HIF-1α and HIF-2α on Notch signaling. It was observed that these two HIF subunits bind to NICD in a competitive manner (Hu et al., 2014). When HIF-1α binds to NICD and Notch-responsive promoters, Notch signaling is activated and cell differentiation is suppressed (Hu et al., 2014), thereby maintaining the undifferentiated cell state in various stem cells and precursor cells (Gustafsson et al., 2005). In contrast, when HIF-2α binds to NICD, Notch signaling is repressed, leading to cell differentiation and stem cell exhaustion (Hu et al., 2014). The coupling of NICD and HIF with the intracellular transducers of niche factor signaling is indeed a common behavior. In TGF-β signaling, for example, both NICD and HIF-1α can bind to the intracellular transducer of TGF-β signals (Blokzijl et al., 2003; Huang Y. et al., 2021), smad3 (mothers against decapentaplegic homolog 3), and the association of HIF-1α and smad3 has been shown to switch the functionality of TGF-β signaling to glycolysis in non-small cell lung cancer (NSCLC) (Huang Y. et al., 2021).

Recently, applying blockade antibodies against PD-1 and its ligand PD-L1 has become a promising strategy for treating advanced cancers (Brahmer et al., 2012; Topalian et al., 2012). The capacity of immune suppression is also one essential feature in the mesenchymal stem cell- (MSC-) based cell therapy (Ankrum et al., 2014; Jiang and Xu, 2020). A growing interest has thus been focused on the interplay of PD-L1 and HIF/Notch/YAP signaling pathways due to the exclusive involvement of HIF/Notch/YAP signaling in the development and homeostasis of stem cells and in the progression of cancers. In particular, YAP can bind to the PD-L1 enhancer region to promote PD-L1 expression, independent of any existing signaling factors and pathways known to upregulate PD-L1, such as EGFR, AKT, MAPK, and interferon- (IFN-) γ (Kim M. H. et al., 2018). In addition to being hypoxic, the TME contains multiple inflammatory factors such as interleukin-6 (IL-6) and remodeling factors such as TGF-β, which can activate signal transducer and activator of transcription 3 (STAT3) to increase the synthesis of HIF-1α and bind to NICD for a synergistic operation on Notch target genes, including the upregulation of PD-L1 expression (Blokzijl et al., 2003; Lee et al., 2009; Yu et al., 2009; Fan et al., 2013; Kitamura et al., 2017; Gupta et al., 2018; Kunnumakkara et al., 2018; Wen et al., 2020). Thus, the pharmaceutical targeting on YAP/Notch/HIF signaling pathways has been proposed as a potential adjunct therapy for cancer treatment, along with the conventional chemotherapy and immune therapy (Janghorban et al., 2018).

## Glossary

AEC2,: anion exchange protein 2
AJ,: adherens junction
AKAP,: A-kinase anchoring protein
ALK5,: activin A receptor type II-like kinase, 53kDa, transforming growth factor β receptor I
AMOT,: angiomotin
Ang II,
angiotensin II
AMPK,: 5′ AMP-activated protein kinase
APC,: adenomatous polyposis coli
aPKC,: atypical protein kinase C
AQP,: aquaporin
ARHGEF,: Rho-guanine nucleotide exchange factor
ARID1A,: AT-rich interactive domain-containing protein 1A
BMK1/Erk5,: big MAP kinase
BMP,: bone morphogenic protein
BNIP,: BCL2/adenovirus E1B 19 kDa protein-interacting protein
βPIX,: p21-activated protein kinase-exchange factor β
CAF,: cancer-associated fibroblast
CaMK2,: Ca^2+^/calmodulin-dependent kinase 2
CaMKP,: CaMK phosphatase
CBP,: cyclic adenosine monophosphate response element-binding protein-binding protein, also named CREB-binding protein (CREBBP)
CK1,: casein kinase 1
CK2,: casein kinase 2
CLASP1,: cytoplasmic linker associated protein 1
CLIP1,: CAP-GLY domain-containing linker protein 1
CREBBP,: cyclic adenosine monophosphate response element-binding protein-binding protein, also named CBP
CSC,: cancer stem cell
CSC-like,: cancer stem cell-like
CSL,: the transcriptional repressor CBF1/suppressor of hairless/Lag-1
CTC,: circulating tumor cell
CTGF,: connective tissue growth factor
Dll1,: Delta-like 1
Dll4,: Delta-like 4
DVL,: Dishevelled
ECM,: extracellular matrix
Ect2,: epithelial cell transforming sequence 2
EGF,: epidermal growth factor (EGF)
EMI,: epithelial-mesenchymal interaction
EMT,: epithelial-mesenchymal transition
ENaC,: epithelial sodium channels
ENO1,: enolase 1
ErK1/2,: extracellular regulated kinases 1/2
ETC,: electron transfer chain
Ex,: Expanded
FAD,: flavin adenine dinucleotide
FADH_2_,: the reduced form of FAD
FAK,: focal adhesion kinase
FBD,: FERM-binding domain
FERM,: 4.1 protein, ezrin, radixin, and moesin
FGF,: fibroblast growth factor
FIH,: the factor inhibiting HIF
FRM6,: FERM domain-containing protein 6
GEF,: guanine-nucleotide exchange factor
GLUT,: glucose transporter
Grb2,: growth factor receptor-bound protein 2
GSK3β,: glycogen synthase kinase 3β
HCC,: hepatocellular carcinoma cell
HIF,: hypoxia-inducible factor
HK,: hexokinase
HPH,: HIF prolyl hydroxylases
HRE,: hypoxia-responsive element
IAP,: integrin-associated protein
IFN,: interferon
IGF-1,: insulin-like growth factor-1
IGF-1R,: IGF-1 receptor
IκBα,: nuclear factor of kappa light polypeptide gene enhancer in B-cells inhibitor, alpha
IL,: interleukin
ILK,: integrin-linked kinase
INF-2,: inverted formin-2
IQGAP1,: IQ motif-containing GTPase activating protein 1
Jag2,: Jagged-2
JaK2,: Janus kinase 2
JNK,: Jun N-terminal kinase
KIBRA,: kidney and brain expressed protein
Keap1,: Kelch-like ECH-associated protein 1
LATS1/2,: large tumor suppressor kinase 1/2
LDHA,: lactate dehydrogenase A
LMW-PTP,: low molecular weight protein tyrosine phosphatase
LOX,: lysyl oxidase
LOXL,: LOX-like enzymes
mAKAP,: muscle-selective A-kinase anchoring protein
MAP4K,: mitogen-activated protein kinase kinase kinase kinase
MAPK,: mitogen-activated protein kinase
MDSC,: myeloid-derived suppressor cell
Merlin,: moesin/ezrin/radixin-like protein
Mito,: mitochondria
MLC,: myosin light chain
MOB1,: Mps one binder (MOB) kinase activator-like 1
MSC,: mesenchymal stem cell
MST1/2,: mammalian Ste20-like protein kinase 1/2
MVB,: multi-vesicular body
MYPT1,: myosin phosphatase target subunit 1
NAD^+^,: nicotinamide adenine dinucleotide
NADP^+^,: nicotinamide adenine dinucleotide phosphate
NADPH,: the reduced form of nicotinamide adenine dinucleotide phosphate
NCX,: sodium-calcium exchanger
NEDD4,: neural precursor cell expressed developmentally downregulated protein 4
NES,: nuclear export signal
NF2,: neurofibromatosis type 2
NF-κB,: nuclear factor kappa-light-chain-enhancer of activated B cells
NICD,: Notch intracellular domain
NLK,: Nemo-like kinase
NLS,: nuclear localization signal
NOS,: nitric oxide synthase
NOX,: NADPH oxidases
Nrf2,: nuclear factor erythroid-derived 2-related factor 2
NRX,: nucleoredoxin
NSCLC,: non–small cell lung cancer
p300,: E1A binding protein p300
PA,: phosphatidic acid
PAK,: P21-activated protein kinase
PALS1,: protein associated with Caenorhabditis elegans Lin-7 protein 1
PAR3,: partition-defective 3
PAR6β,: partition-defective 6β
PATJ,: PALS1-associated tight junction protein
PD-1,: programmed death-1
PDE,: phosphodiesterase
PDGF,: platelet-derived growth factor
PDK,: pyruvate dehydrogenase kinase (PDK)
PD-L1,: programmed death ligand-1
PDZ,: Post-synaptic density 95, Discs large, and Zonula occludens-1
PHD: prolyl hydroxylase domain-containing proteins
PID,: protein interaction domain
PI3K,: phosphatidyl-inositol-3-kinase
PIP_2_,: phosphatidylinositol (4,5)-bisphosphate
PIP_3_,: phosphatidylinositol (3,4,5)-trisphosphate
PIX,: p21-activated protein kinase exchange factor
PKA,: cAMP-dependent protein kinase
PKB,: protein kinase B
PKC,: protein kinase C
PKG,: cGMP-dependent protein kinase
PKM2,: pyruvate kinase isozymes M2
Poldip2,: polymerase δ-interacting protein 2
POPX2,: partner of PIX 2, also known as protein phosphatase 2C-like domain-containing metal-dependent protein phosphatase (PPM), PPM1F, or CaMK phosphatase (CaMKP)
PP2AC,: PP2A catalytic subunit
PPM,: protein phosphatase 2C-like domain-containing metal-dependent protein phosphatase
PPM1A,: protein phosphatase, Mg^2+^/Mn^2+^-dependent 1A
PPM1F,: protein phosphatase, Mg^2+^/Mn^2+^-dependent 1F
PPP,: protein serine/threonine phosphatases
PTEN,: phosphatase and tensin homolog
PTK: protein tyrosine kinase
PTM,: post-translational modification
PTP,: protein tyrosine phosphatases
PTPN14,: protein tyrosine phosphatase non-receptor type 14
PX,: pbox consensus sequence
Pyk2,: proline-rich tyrosine kinase 2
Rac1,: Ras-related C3 botulinum toxin substrate 1
RASSF,: Ras-association domain family
RBPJ,: recombination signal-binding protein for immunoglobulin κJ region
Ref-1,: redox factor-1
RhoA,: Ras homolog family member A
ROCK,: Rho-associated protein kinase
RyR,: ryanodine receptor
SARAH,: SAV/RASSF/HPO
SAV1,: Salvador family WW domain-containing protein 1
SCF,: Skp1-Cullin-1-F-box protein
SCF^β-TrCP^,: SCF type of E3 ubiquitin ligase
SCLC,: small cell lung cancer
SH2,: Src homology 2
SHP,: SH2 domain-containing protein tyrosine phosphatase
SHPS-1,: SHP substrate-1
SIAH1/2,: seven in absentia homolog 1/2
SLMAP,: sarcolemmal membrane-associated protein
Smad3,: mothers against decapentaplegic homolog 3
SNF,: sucrose non-fermentable
SR,: sarcoplasmic reticulum
Src,: steroid receptor coactivator
SrcFK,: Src family kinase
STAT3,: signal transducer and activator of transcription 3
STK25,: serine/threonine kinase 25
STRIP1/2,: STRN-interacting protein 1/2
STRIPAK,: STRN-interacting phosphatase and kinase
STRN,: striatin
SWH,: Salvador–Warts–Hippo
SWI,: switch
TAZ,: transcriptional coactivator with PDZ-binding motif
TE,: trophectoderm
TEAD,: the transcriptional enhancer factor TEF-1, TEC1, and AbaA (TEA) domain
TGFβ,: transforming growth factor-β
TGFβR,: transforming growth factor-β receptor
TJ,: tight junction
TME,: tumor microenvironment
TNF,: tumor necrosis factor
TRAF4,: TNF receptor-associated factor 4
TRP,: transient receptor potential
VEGF,: vascular EGF
VEGFR,: VEGF receptor
VHL,: von Hippel–Lindau tumor suppressor protein E3 ubiquitin ligase
WASP,: Wiskott–Aldrich syndrome protein
WAVE1,: WASP family verprolin homologous protein 1
WWC,: WW domain and C2 domain-containing protein
WWTR1,: WW domain-containing transcription regulator 1
X-ROS,: NOX-derived ROS
YAP,: Yes-associated protein
Yes,: Src family, non-receptor proto-oncogene tyrosine-protein kinase.
